# Score-Driven Modeling of Spatio-Temporal Data

**DOI:** 10.1080/01621459.2021.1970571

**Published:** 2021-10-04

**Authors:** Francesca Gasperoni, Alessandra Luati, Lucia Paci, Enzo D’Innocenzo

**Affiliations:** a MRC Biostatistics Unit, University of Cambridge, Cambridge, UK; b Department of Statistical Sciences, University of Bologna, Bologna, Italy; c Department of Statistical Sciences, Università Cattolica del Sacro Cuore, Milano, Italy

**Keywords:** fMRI, Multivariate Student-t distribution, Robust filtering, SAR models, Spontaneous, activations

## Abstract

A simultaneous autoregressive score-driven model with autoregressive disturbances is developed for spatio-temporal data that may exhibit heavy tails. The model specification rests on a signal plus noise decomposition of a spatially filtered process,where the signal can be approximated by a nonlinear function of the past variables and a set of explanatory variables, while the noise follows a multivariate Student-t distribution. The key feature of the model is that the dynamics of the space-time varying signal are driven by the score of the conditional likelihood function.When the distribution is heavy-tailed, the score provides a robust update of the space-time varying location. Consistency and asymptotic normality ofmaximum likelihood estimators are derived along with the stochastic properties of the model. The motivating application of the proposed model comes from brain scans recorded through functional magnetic resonance imaging when subjects are at rest and not expected to react to any controlled stimulus. We identify spontaneous activations in brain regions as extreme values of a possibly heavy-tailed distribution, by accounting for spatial and temporal dependence.

## 1 Introduction

Accounting for dependence across space and time represents the key ingredient in analyzing spatially and temporally referenced data. We are concerned with spatio-temporal datasets that are available in the form of areal or regional data, that is, data recorded on a partition of the spatial domain of interest. Examples of spatio-temporal data collected over discrete domains arise in many research fields; see, for example, Waller et al. (1997) on disease mapping, Gelfand et al. (1998) on real estate, Vivar and Ferreira (2009) with an application to crime data, Hooten and Wikle (2010) on epidemic studies, Blasques et al. (2016) on financial systemic risk, Paci et al. (2017) on remote sensing imaging. In settings where both space and time are discrete, there is a rich literature on spatiotemporal modeling based on a Gaussian Markov random field (GMRF) structure (Rue and Held 2005), generally from a Bayesian perspective. In this framework, spatial random effects are usually defined through full conditional distributions in the form of conditionally autoregressive (CAR) specifications (Waller et al. 1997; Gelfand et al. 1998; Vivar and Ferreira 2009) that enable direct Markov chain Monte Carlo fitting. Differently, they are assumed to be generated from a stochastic partial differential equation spatial process (Lindgren, Rue, and Lindström 2011), constructed as a discretization of a Matèrn Gaussian field.

In this work, we take an alternative avenue and propose a novel observation-driven approach to model discrete spatiotemporal data. The model belongs to the class of score-driven models for time-varying parameters, introduced in the context of volatility estimation and originally referred to as generalized autoregressive score models (GAS; Creal, Koopman, and Lucas 2013) or dynamic conditional score models (DCS; Harvey 2013). The key feature of score-driven models is that the dynamics of time-varying parameters are driven by the score of the conditional likelihood, taken with respect to the parameters themselves. In our case, the distribution is Student-*t* and the location is the space-time varying parameter. When the distribution is heavy-tailed, the driving score provides a more robust update of the space-time varying location since it has thinner tails compared to the Normal case. In particular, the smaller the degrees of freedom parameter the more robust the filter is; conversely, when the degrees of freedom increase to infinity, the score of the Student-t distribution converges to that of a Gaussian distribution. With respect to the existing score-driven models, the one developed in this article can be interpreted as a spatial extension of the score-driven filter for signal extraction introduced in the univariate case by Harvey and Luati (2014) and considered by D’Innocenzo, Luati, and Mazzocchi (2020) in a purely dynamic and low-dimensional setting.

The spatial score-driven model developed in this article is based on the family of spatial autoregressive (SAR) models. SAR models serve as the workhorse of spatial regression modeling, particularlyin the spatial econometrics literature (Anselin 1988; LeSage and Pace 2009; Robinson and Rossi 2015). Related to our analysis, recent examples of SAR models include neuroimaging (Messé et al. 2015) and extremes of areal data (Fix, Cooley, and Thibaud 2021). With respect to the GMRF approach, SAR models are well suited to maximum likelihood estimation, thus offering a natural setting for extending score-driven models to spatio-temporal data. In particular, we assume that the score drives the evolution of the signal of a spatially filtered multivariate dataset with spatially dependent errors. In other words, we specify a model by using the first-order simultaneous autoregressive process with autoregressive disturbances, in short SARAR(1,1). The SARAR specification (Kelejian and Prucha 2010; Lee and Yu 2010; Catania and Billé 2017) is fairly general since it allows for spatial dependence in the response variables, in the explanatory variables as well as in the disturbances. This makes our method different from the recent works on score-driven models within the spatial regression setting, such as Blasques et al. (2016) and Billé, Blasques, and Catania (2019), who exploited the score-driven framework to update time-varying weight matrices to account for dyamic volatility in spatio-temporal models.

The motivating application of the proposed model is the study of functional magnetic resonance imaging (fMRI) data. Functional magnetic resonance imaging is a noninvasive technique that measures the increase in the oxygenation level at some specific brain regions, as long as an increase in blood flow occurs, due to some brain activity. The latent signal in the observed fMRI data is referred to as the blood oxygenation leveldependent (BOLD) signal, see Lindquist (2008) for a general introduction to fMRI.

When analyzing fMRI data, major inferential challenges arise due to their complexity (Guindani and Vannucci 2018). As a matter of fact, fMRI are recorded as time series, observed at different brain regions of interests or, on a finer scale, at different voxels, across individuals. The crucial role of spatial and temporal dependence in fMRI data is acknowledged by a large stream of the literature so that spatio-temporal models have been extensively investigated for the analysis of functional connectivity in single- and multi-subject studies; see, among others, Smith and Fahrmeir (2007), Kang et al. (2012), Zhu, Fan, and Kong (2014), Zhang et al. (2016), and Mejia et al. (2020).

These approaches are mainly designed to deal with the analysis of task-based experiments, where the data are recorded in response to some external stimulus and the goal is detecting those regions that are activated in response to the stimulus. On the other hand, in the recent years, the interest has been concentrating toward resting state fMRI (R-fMRI) sessions, that do not require subjects to perform any specific task. R-fMRI signals have been shown to relate to the spontaneous neural activity that refers to brain activity not attributed to any experimental conditions, neither to other specific inputs, that is, it represents the neuronal activity that is intrinsically generated by the brain (Fox and Raichle 2007). This article addresses the main challenge associated with the analysis of R-fMRI data, that is the detection of brain spontaneous activations.

In a seminal work on detecting activations in task-based fMRI data, Worsley (2003) referred to an activation as “a local increase in the effect of the task, with most of the brain unaffected by the task” and observed that the problem of detecting activations has much in common with outlier detection. The author assumed temporally correlated Gaussian errors, fitted a linear regression model separately for each brain region and then performed spatial smoothing. Turning to R-fMRI data, Wang et al. (2008) aimed at investigating the existence of spontaneous activity in the primary visual areas, yet based on the assumption that the noise affecting the BOLD signal is Gaussian. In both cases, spontaneous activations are identified as points exceeding some threshold, typically twice the estimated standard deviation. Though often adequate to task-based experiments, the Gaussian assumption may turn out to be restrictive in R-fMRI data, where, ideally, no exogenous stimulus affects the underlying signal and the noise dynamics reflect the human brain resting activity (Fox and Raichle 2007). The results of the extensive analysis presented by Eklund, Nichols, and Knutsson (2016) questioned the validity of a large number of fMRI studies and opened a debate in the field of neuroimaging. According to the authors, the main cause of invalid results is that fMRI data do not usually follow the assumed Gaussian shape. With this in mind, a more flexible modeling framework, encompassing the Gaussian case, is advocated.

In this article, we move a step forward and develop a procedure for detecting spontaneous activations based on the assumption that they correspond to extreme values of a possibly heavy-tailed distribution. The spatio-temporal score-driven model introduced in the article delivers robust estimates of the underlying BOLD signal and leaves in the residuals, the one-step-ahead spatial prediction errors, the information on spontaneous activations. A procedure for identifying brain activations thus rests on the analysis of extreme quantiles in the residuals of the estimated model.

Summarizing, the main contribution of the article is twofold. The theoretical contribution consists in the specification of a spatio-temporal model for analyzing several heavy-tailed time series that are spatially correlated. The Student-t SAR score-driven model with explanatory variables and SAR disturbances developed in the article lends itself to a number of potential applications and nests several models commonly applied in the spatial and in the time series literature, including, among others, the Gaussian SARAR model of Anselin (1988) and the nonlinear filter of Harvey and Luati (2014). Likelihood-based theory is developed to provide model estimation by the method of maximum likelihood. Consistency and asymptotic normality of maximum likelihood estimators (MLE) are proved for large T (number of time series observations) and fixed R (number of spatial regions). In a linear Gaussian framework, a similar setting is considered by Yu, de Jong, and Lee (2008), Korniotis (2010) and Gupta and Robinson (2015), where the properties of MLE and Quasi-MLE (QMLE) for spatial or panel data are derived for large T and different assumptions on R, encompassing the case when it is fixed. In contrast with the aforementioned articles, in our setting, serial dependence is captured by the space-time location that, due to the Student-*t* score-driven updating mechanism, is a nonlinear function of the past. The asymptotic theory developed in the article thus extends to spatial models the recent results on maximum likelihood estimation in nonlinear observation-driven models derived by Blasques et al. (2020), in the univariate case, and by D’Innocenzo, Luati, and Mazzocchi (2020), in the dynamic location case. A side contribution to large-T asymptotics in likelihood theory for nonlinear dynamic spatial models is the analytic expression of the conditional information matrix, available in closed-form and valid for the specifications nested in the spatial score-driven model with fixed effects developed in the article.

On the applied side, we build a model-based procedure for detecting spontaneous activations in R-fMRI data based on a robust spatio-temporal score-driven filter. Specifically, activations are identified as extreme values of (possibly) heavy-tailed residuals obtained from a robust procedure for signal extraction. A weighted anatomic distance between brain regions is also designed to account for the spatial structure of the data. The model is applied to study multi-subject brain imaging data coming from a pilot study of the Enhanced Nathan Kline Institute Rockland Sample project.

The article is organized as follows. In Section 2 we introduce the spatial score-driven model and discuss its properties. Model estimation and asymptotic results are provided in Section 3, together with a summary of an extensive simulation study. Section 4 illustrates the results of the analysis performed on the R-fMRI data, with emphasis on the specification of the spatial weight matrix and the identification of brain spontaneous activations. Section 5 concludes the article and supplementary materials complement it.

## 2 Spatial Score-Driven Modeling

### 2.1 Model Developments

Let y_t_ = (*y_1_*,*_t_*,…,*y_R_*,*_t_*)ᵀ be a *R*-dimensional vector of time series observed at time *t*,*t* = 1,…, *T*,where *T* is the length of each time series and *R* is the number of spatial regions. The spatial score-driven model is based on the decomposition of a spatial autoregressive signal adjusted for fixed effects plus a spatial autoregressive noise, that is

(1) $$\begin{array}{ll} y_{t}= & \rho_{1}W_{1}y_{t}+{\text{X}}_{t}\beta +\mu_{t}+\varepsilon_{t},\\ \varepsilon_{t}= & \rho_{2}W_{2}\varepsilon_{t}+\eta_{t}, \end{array}$$

where *ρ*_1_ and *ρ*_2_ are the spatial autocorrelation parameters, **W**_1_ and **W**_2_ are the *R*×*R* spatial weight matrices,**X**_t_ is an *R*×(*p*+1) matrix containing *p* nonstochastic, exogenous, regressors and an intercept, β is a (*p* + 1)-dimensional vector of unknown coefficients, μt = (μ_1,*t*_,…, μ*_R,t_*)ᵀ is the temporal signal, εt = (ε1,*_t_*,…, ε*_R,t_*)ᵀ is the vector of spatial (first-order) autoregressive error terms and the noise η_t_ = (η_1,*t*_,…, η_*R*,*t*_)ᵀ follows a multivariate Student-*t* distribution with v > 0 degrees of freedom and diagonal shape matrix Λ = diag (*e^2λ^_1_*,…, *e^2λ^_R_*), that is, $\eta_{t}\overset{\text{iid}}{∼}t_{v}(0,\text{Λ})$.

Equation (1) can be written as

$$\begin{array}{ll} Z_{1}y_{t}= & {\text{X}}_{t}\beta +\mu_{t}+\varepsilon_{t}\\ Z_{2}\varepsilon_{t}= & \eta_{t}, \end{array}$$

where **Z**_1_ = **I**_R_ − *ρ*_1_**W**_1_ and **Z**_2_ = **I**_R_ − *ρ*_2_**W**_2_ are spatial filtering matrices and **I**_R_ is the *R* × *R* identity matrix. Note that matrices **Z**_1_ and **Z**_2_ depend on the unknown parameters *ρ*_1_ and *ρ*_2_, respectively, but they are static, as neither **W**_1_,**W**_2_ nor *ρ*_1_, *ρ*_2_ depend on time. The spatial dependence parameter *ρ*_1_ captures the impact of the spatially weighted contemporaneous-dependent variables**W**_1yt_ on *y_t_* while *ρ*_2_ describes the impact of spatially weighted disturbances **W**__2_εt_ on ε_t_. When the matrices **Z**_1_ and **Z**_2_ are invertible, Equation (1) can be written in reduced form, that is,

(2) $$\begin{array}{ll} y_{t}= & Z_{1}^{-1}{\text{X}}_{t}\beta +Z_{1}^{-1}\mu_{t}+Z_{1}^{-1}\varepsilon_{t},\\ \varepsilon_{t}= & Z_{2}^{-1}\eta_{t}. \end{array}$$

Here, we consider spatial weight matrices **W**_1_ and **W**_2_ that are row-stochastic, that is, $\sum_{j=1}^{R}w_{1,ij}=\sum_{j=1}^{R}w_{2,ij}=1$ and with null diagonal elements, that is, **W**_1_,*_ii_* = **W**_2_,*_ii_* = 0 for *i* = 1,…, *R*, by construction. As such, **W**_1_ and **W**_2_ are not symmetric but with all the eigenvalues less than or equal to 1 in modulus. As a consequence, **Z**_1_ and **Z**_2_ are nonsingular for all values of |*ρ*_1_| < 1 and |*ρ*_2_| < 1, respectively; invertibility of **Z**_1_ and **Z**_2_ guarantees the convergence of the von Neumann sum, that is, the reduced form in Equation (2) is valid. Often, it is set **W**_1_ = **W**_2_ = **W**, see Kelejian and Prucha (2010).

To facilitate the spatial interpretation, we can rewrite the model by using the infinite series expansion as in LeSage and Pace (2009), that is,

(3) $$y_{t}=\sum_{k=0}^{\infty }{\left(\rho_{1}W_{1}\right)}^{k}X_{t}\beta +\sum_{k=0}^{\infty }{\left(\rho_{1}W_{1}\right)}^{k}\mu_{t}+\left(\sum_{k=0}^{\infty }{\left(\rho_{1}W_{1}\right)}^{k}\right)\left(\sum_{k=0}^{\infty }{\left(\rho_{2}W_{2}\right)}^{k}\right)\eta_{t}.$$

Equation (3) reveals the simultaneous nature of the spatial autoregressive process that relates all the locations in the system, producing the so called global spillover effect (Anselin 2003). Specifically, if **W**_1_ and **W**_2_ correspond to first-order neighbors, then their powers involve higher order neighbors, so that any region is affected by all the others. However, the powers of the autoregressive parameters (with |*ρ*_1_| < 1, |*ρ*_2_| < 1) ensure that the spillover effect decreases with higher orders of neighbors so that closer regions are more affected than far ones.

Conditionally on the past information set, 𝓕_*t−1*_ = σ{**y**_*t−1*_,**y**_*t−2*_,…} that is the filtration of the process at time *t*−1, we have that $Z_{1}^{-1}Z_{2}^{-1}\eta_{t}=y_{t}-Z_{1}^{-1}{\text{X}}_{t}\beta -Z_{1}^{-1}\mu_{t}$ is distributed as a zero mean Student-*t* random vector with variance-covariance matrix equal to $\Sigma =v/(v-2)Z_{1}^{-1}Z_{2}^{-1}\Lambda {\left(Z_{2}^{-1}\right)}^{⊤}{\left(Z_{1}^{-1}\right)}^{⊤}$, for *v* > 2. Assuming that the conditional mean of the data is 𝓕_*t−1*_ - measurable, that is, $E\left(y_{t}|{ℱ}_{t-1}\right):=Z_{1}^{-1}{\text{X}}_{t}\beta +Z_{1}^{-1}\mu_{t|t-1}$,we may write

(4) $$y_{t}|{ℱ}_{t-1}\sim t_{v}(Z_{1}^{-1}{\text{X}}_{t}\beta +Z_{1}^{-1}\mu_{t|t-1},Z_{1}^{-1}Z_{2}^{-1}\Lambda {\left(Z_{2}^{-1}\right)}^{⊤}{\left(Z_{1}^{-1}\right)}^{⊤}).$$

Without specifying any distributional assumption for **μ**_*t*_, *a* stochastic recurrence equation can be set up to approximate the path of **μ**_*t*_ based on the past observations. The subscript notation *t* | *t* − 1 is used to emphasize the fact that μ_t_|*t*-1 = 𝔼(**μ**_*t*_ | 𝓕_*t−1*_) is an approximation of the dynamic location process at time *t*, which becomes predictable given the past. In particular, we specify a score-driven filter as follows,

(5) $$\mu_{t+1|t}=\phi \mu_{t|t-1}+Ku_{t},$$

where φ is a temporal autoregressive parameter, **K** = diag(κ_1_,…, K*R*) is a diagonal matrix of coefficients, and

(6) $$u_{t}=Z_{2}Z_{1}v_{t}/\alpha_{t},$$

where $v_{t}=y_{t}-Z_{1}^{-1}{\text{X}}_{t}\beta -Z_{1}^{-1}\mu_{t|t-1}$is the one-step-ahead prediction error of the model written in reduced form and

(7) $$\alpha_{t}=1+{\left(y_{t}-Z_{1}^{-1}{\text{X}}_{t}\beta -Z_{1}^{-1}\mu_{t|t-1}\right)}^{⊤}Z_{1}^{⊤}Z_{2}^{⊤}\Lambda^{-1}Z_{2}Z_{1}\left(y_{t}-Z_{1}^{-1}{\text{X}}_{t}\beta -Z_{1}^{-1}\mu_{t|t-1}\right)/\nu .$$

The key feature of score-driven models is that the driving force in the dynamic equation for the time-varying parameter, that is, ut in Equation (6), is proportional to the score of the conditional likelihood of the time-varying parameter of interest, that in our case is the space-time varying signal, μ_*t*|*t*-1_. As a matter of fact,

$$\begin{array}{ll} \frac{\partial \ln f\left(y_{t}|{ℱ}_{t-1}\right)}{\partial \mu_{t|t-1}} & \\ =\frac{\nu +R}{\nu }Z_{2}^{⊤}\Lambda^{-1} & Z_{2}Z_{1}\left(y_{t}-Z_{1}^{-1}{\text{X}}_{t}\beta -Z_{1}^{-1}\mu_{t|t-1}\right)/\alpha_{t}\\ =\frac{v+R}{v}Z_{2}^{⊤}\Lambda^{-1} & u_{t} \end{array}$$

where

(8) $$\begin{array}{l} f\left(y_{t}|{ℱ}_{t-1}\right)=\frac{\Gamma \left(\frac{v+R}{2}\right)}{\Gamma \left(\frac{v}{2}\right){\left(\pi v\right)}^{R/2}}{\left|Z_{1}^{-1}Z_{2}^{-1}\Lambda {\left(Z_{2}^{-1}\right)}^{⊤}{\left(Z_{1}^{-1}\right)}^{⊤}\right|}^{-1/2}\\ (1+{\left(y_{t}-Z_{1}^{-1}{\text{X}}_{t}\beta -Z_{1}^{-1}\mu_{t|t-1}\right)}^{⊤}Z_{1}^{⊤}Z_{2}^{⊤}\Lambda^{-1}Z_{2}Z_{1}\\ {\left(y_{t}-Z_{1}^{-1}{\text{X}}_{t}\beta -Z_{1}^{-1}\mu_{t|t-1}\right)/v)}^{-\frac{v+R}{2}} \end{array}$$

is the density of the conditional Student-*t* distribution. The rationale behind the recursion (5) is very intuitive. Analogously to the Gauss–Newton algorithm, it improves the model fit by pointing in the direction of the greatest increase of the likelihood. A detailed discussion on optimality of score-driven updates is given by Blasques, Koopman, and Lucas (2015).

We refer to the set of Equations (4)–(7) as the spatial score-driven model in reduced form. This specification has the following implications. The first implication is that ut is a martingale difference sequence by construction, that is, **μ**_*t*_|𝓕_*t−1*_ = 0, which follows by the properties of the score. As such, it plays the role of the driving force in observation-driven models, where the dynamics of the time-varying parameters depend on a nonlinear function of past observations. The second implication is that when υ → ∞, then *u_t_* converges to the spatial one-step-ahead prediction error, **Z**_2_**Z**_1_*v_t_* = **Z**_2_**Z**_1_*y_t_* − **Z**_2_**X**_t_*β* − **Z**_2_μ_*t*|*t*.1_, and both *u_t_* and *v_t_* will have Gaussian distribution as well as *y_t_*. Hence (i), a linear Gaussian (spatial autoregressive) model is encompassed by our specification and (ii) when the data come from a heavy-tailed distribution, then Equation (5) delivers a robust filter in the sense of Calvet, Czellar, and Ronchetti (2015, prop. 1). Indeed, the positive factors *α_t_* in Equation (6) are scalar weights that involve the Mahalanobis distance. They possess the role of downsizing large deviations from the mean incorporated in the conditional Student-*t* prediction error *v_t_*. In turn, unless υ → ∞, the driving force ut in Equation (6) has a thin tails distribution, since it can be written as *u_t_* = **Z**_2_**Z**_1_*v_t_* (1−*b_t_*) where *b_t_* = 1 − 1/*α_t_* is a Beta $\left(\frac{R}{2},\frac{\nu }{2}\right)$ distributed random variable, as

(9) $$b_{t}=\frac{{\left(Z_{2}Z_{1}v_{t}\right)}^{⊤}\Lambda^{-1}\left(Z_{2}Z_{1}v_{t}\right)/v}{1+{\left(Z_{2}Z_{1}v_{t}\right)}^{⊤}\Lambda^{-1}\left(Z_{2}Z_{1}v_{t}\right)/v},$$

see Kotz and Nadarajah (2004, pp. 19) or Harvey (2013, prop. 39). In practice, ut is a winsorized version of **Z_2_1Z**_1_*v_t_*. As such, extremes are cut off from *u_t_*, and, consequently, from μ_*t*|*t*-1_, while vt conveys the information on extreme values or outliers affecting the data. The smaller the degrees of freedom parameter v, the more robust the filter is, that is, the less sensitive the space-time varying signal is to outliers, and, with a different perspective, the more informative the prediction error is about anomalous observations. The diagonal elements of the matrix **K** in Equation (5), that is, the coefficients κ_r_, further regulate the impact of ut on the filtered signal *μ_t+1|t_*.

In summary, when the data come from a heavy-tailed distribution, ut is less sensitive to extreme values than the score of a Gaussian distribution. Conversely, if the data-generating process is normal, or, in our setting, if υ → ∞, then the score of the Student-1 distribution converges to that of a Gaussian distribution. Discarding the spatial components, that is, *ρ*_1_ = = 0, while keeping υ → ∞, gives a linear Gaussian signal plus noise model. Ignoring the dynamics, spatial error models (SEM) and SAR models with fixed effects are recovered when *ρ*_1_ = 0 or *ρ*_2_ = 0, respectively.

### 2.2 Convergence to the Multivariate Gaussian Distribution

A relevant issue when moving from the univariate Student-1 distribution to its multivariate counterpart is concerned with the rate of convergence of the multivariate Student-t to the Gaussian distribution, as long as the degrees of freedom increase toward infinity. The following proposition shows that the convergence rate depends on R, the spatial (or panel or cross section) dimension: the larger R, the slower the convergence to the Normal.

#### Proposition 1

The following asymptotic expansion is valid, when υ → ∞, for *f* (*y_t_*), the *R*-variate Student-*t* density with zero mean, unit scale and υ degrees of freedom,

$$f\left(y_{t}\right)=\phi \left(y_{t}\right)\left(1+\frac{{\left(y_{t}^{\prime }y_{t}\right)}^{2}-2Ry_{t}^{\prime }y_{t}+R(R-2)}{4v}+O\left(\frac{1}{v^{2}}\right)\right)$$

where φ(yt) is the multivariate standard normal density.

A related, practical, aspect regards determining, for any fixed R, a finite value of υ_0_ such that for υ ≥ υ_0_ one can refer to the Normal distribution. It is well known that, in the univariate case, the value υ_0_ = 30 is taken as a bound for relying to the Normal approximation (Fisher 1925). Note that, for *R* = 1, the above term in υ^−1^ collapses to the first term in Fisher’s expansion, where termwise integration is carried over to measure the size of the approximation. The proof of the proposition, in Section S1, shows that the term involving *R^2^*/υ comes from the integration constant, which actually requires υ ≥ υ_0_ where υ_0_ = 30*R^2^* to reliably approach the Gaussian integration constant. The latter value encompasses the univariate case, when *R* = 1 and υ_0_ = 30. Note, however, that depending on *y_t_*, the actual value of υ for approaching normality may be smaller than υ_0_, as the density kernel expansion plays a role as well, see the discussion in Section S1.1.

## 3 Maximum Likelihood Estimation

The model specified by Equations (*4*)-(*7*) depends on a set of static parameters, that we collect in a vector θ ∈ Θ. To set up the notation, let us sort the elements of the matrices Λ and **K** in the vectors λ and *K* = diag{**K**}, with generic elements λ_r_ and κ_r_, for *r* = 1,… *R*, respectively. The vector of static parameters to be estimated is then $\theta ={\left(\beta^{⊤},\rho_{1},\rho_{2},v,\lambda^{⊤},\phi ,\kappa^{⊤}\right)}^{⊤}$, with $\theta \in \Theta \subset {\mathbb{R}}^{2R+p+5}$, where β are the *p*+1 regression coefficients, *ρ*_1_ and *ρ*_2_ are the spatial parameters, the pair (υ, λᵀ)ᵀ characterizes the Student-t conditional distribution ofyt given the past and (φ, Kᵀ)ᵀ are the parameters that determine the dynamic evolution of the conditional location μ*t*|*t*−1.

Estimation is carried out by the method of maximum likelihood. Once estimated the parameters and fixed an initial condition for the stochastic recurrence Equation (5), the timevarying signal of the spatially filtered data can be obtained by a simple recursion. For inference to be valid asymptotically, besides the usual regularity conditions typical of maximum likelihood estimation, itisrequired thatthe processthathas generated the observations yt has some stochastic properties such as stationarity, ergodicity and bounded unconditional moments. It is also required, for filtering purposes, that the initial conditions selected for starting the recursion are asymptotically negligible, that is, the filter is invertible, see Blasques et al. (2018) for a discussion of invertibility in nonlinear models. In essence, it is required that the recursion ${\hat{\mu }}_{t|t-1}$ that corresponds to a fixed initial value μ_1|0_ converges exponentially fast almost surely (e.a.s.) to a unique stationary ergodic sequence {μ*t*|*t*−1}_*t*∈𝕫_. Propositions 2 and 3 state the conditions under which these properties hold in the current setting. Theorems 3.1 and 3.2 then establish consistency and asymptotic normality of the MLE of θ. Proofs are reported in the Supplementary materials.

### Proposition 2

Let us consider the model specified by Equation (1) with $||X_{t}||<\infty ,\eta_{t}\overset{\;\text{iid}\;}{\sim }t_{v}(0,\Lambda ),v>0,\left|\rho_{1}\right|<1,\left|\rho_{2}\right|<1$ and **W**_1_ and **W**_2_ row-stochastic. Let us assume that $\mu_{t+1}=\phi \mu_{t}+K\eta_{t}/\left(1+\eta_{t}^{⊤}\Lambda^{-1}\eta_{t}/\nu \right)$, with |φ| < 1 and **K** positive definite. Then, the sequence {**y**_*t*_}_*t*∈𝕫_ is strictly stationary and ergodic. More over, if ||X_*t*_||^*n*^ < 221E;, for n > 0 and $\lim_{T\to \infty }\frac{1}{T}X_{t}^{T}X_{t}$ exists and is non singular, then $E\left({\left\|y_{t}\right\|}^{n}\right)<\infty ,\forall n\geq v$.

### Proposition 3

Let us assume that Proposition 2 holds and, in addition, that the filter in Equation (5) is contractive, that is, $E\left({\mathrm{logsup}}_{\theta \in \Theta }\left\|{∐}_{k=1}^{j}X_{k-j+1}\right\|\right)<0$, where $X_{t}=\partial \mu_{t+1|t}/\partial \mu_{t|t-1}^{⊤}$. Then, sup$\sup_{\theta \in \Theta }\left\|{\hat{\mu }}_{t|t-1}-\mu_{t|t-1}\right\|\overset{\text{e}.\text{d}.\text{s}.}{\to }0\;\text{as}\;t\to \infty$. Furthermore, $\sup_{t}E\left(\sup_{\theta \in \Theta }{\left\|{\hat{\mu }}_{t|t-1}\right\|}^{n}\right)<\infty$ and $E\left(\sup_{\theta \in \Theta }{\left\|\mu_{t|t-1}\right\|}^{n}\right)<\infty$.

We now focus on the estimation of *θ*. Let us denote as 𝓁_t_ (*θ*) the contribution to the log-likelihood of the *t*th observation *y_t_* based on the stationary solution μ*t*|*t*−1, that is, the logarithm of the conditional density in Equation (8) as a function of θ and, for the whole sample,$\ell_{T}(\theta )=\sum_{t=1}^{T}\ell_{t}(\theta )$. Let us define the empirical likelihood ${\hat{\ell }}_{t}(\theta )$ as the logarithm of the conditional density in Equation (8) evaluated at ${\hat{\mu }}_{t|t-1}$ and, analogously, ${\hat{\ell }}_{T}(\theta )=\sum_{t=1}^{T}{\hat{\ell }}_{t}(\theta )$. The maximum likelihood estimator of *θ* is then ${\hat{\theta }}_{T}=\arg\max_{\theta \in \Theta }{\hat{\ell }}_{T}(\theta )$. Note that invertibility of the filter (Proposition 3) ensures that no matter the specific value of *μ*1|0, the likelihood *𝓁_t_*(*θ*) is uniquely approximated by ${\hat{\ell }}_{t}(\theta )$. We also assume that the model is correctly specified in that μ*t*|*t*−1 = μ*t*|*t*−1 (θ_0_) where θ_0_ is the true parameter value.

### Theorem 3.1

Let us consider the model specified by Equations (4)-(7) with **W**_1_ and **W**_2_ row-stochastic, |*φ*| < 1, |*ρ*_1_| < 1, |*ρ*_2_| < 1, υ > 0, κ_r_ > 0, *r* = 1,…, *R*. Let Propositions 2 and 3 hold. In addition, let us assume that the true parameter vector θ_0_ lies in the interior of the compact space Θ and that ∀θ ∈ Θ, if θ ≠ θ_0_, then μ*t*|*t*−1(θ) ≠ μ*t*|*t*−1(θ0) almost surely ∀_*t*_ ≥ 1. Then ${\hat{\theta }}_{T}\to_{\text{a}.\text{s}.\;}\theta_{0}$

### Theorem 3.2

Let us assume that the conditions of Theorem 3.1 hold. In addition, let 𝔼[||**𝓧**_t_ ⊗ **𝓧**_11_|] < 1. Then, $\sqrt{T}({\hat{\theta }}_{T}-\theta_{0})\Rightarrow N\left(0,ℐ{\left(\theta_{0}\right)}^{-1}\right)$, where $ℐ\left(\theta_{0}\right)=E\left[{ℐ}_{t}(\theta )\right]$ and ${ℐ}_{t}(\theta )$ is the conditional information matrix reported in Section S6.

Consistency (Theorem 3.1) and asymptotic normality (Theorem 3.2) are proved for large *T* and fixed *R*, thus extending to spatial models the recent results on maximum likelihood estimation in nonlinear observation-driven models derived by Blasques et al. (2020), in the univariate case, and by D’Innocenzo, Luati, and Mazzocchi (2020), in the dynamic location case (*ρ*_1_ = 0, *ρ*_2_ = 0 without fixed effects). In non Gaussian dynamic models, the closed-form expression of the Fisher information matrix is typically prohibitive, see, for instance, Fiorentini, Sentana, and Calzolari (2003). Inference on θ0 can be carried out based on the conditional information matrix, available in analytic form (see Section S6 and the discussion therein). On the other hand, in Gaussian models, the conditional information matrix coincides with the Fisher information matrix. Indeed, for υ → ∞, the recursions that lead to the conditional information matrix of the spatial score-driven model collapse to the formulae for the Fisher information matrix derived by Anselin (1988, pp. 64–65) in the Gaussian SARAR model (υ → ∞, φ = 0, **K** = 0).

In practice, we compute the maximum likelihood estimates via numerical optimization techniques that are suitable for nonlinear functions. In particular, we use the R function nlminb. The associated R code is also available as supplementary materials.

### 3.1 Simulation Study

An extensive simulation study is carried out to assess (a) the finite sample properties of the MLE, (b) the impact of different exogenous variables, (c) the effects of potential misspecification. All the details of the simulation design, composed of a total of 26 different scenarios, are deferred to Section S7, along with a discussion of the results. In synthesis, we may summarize the results as follows: (a) the true values of φ, *ρ*_1_, *ρ*_2_, and λ are always very well recovered, while the degrees of freedom parameter is typically slightly underestimated. Conversely, the estimates of κ are sometimes moderately overestimated, with increasing precision as v increases; (b) adding exogenous variables does not alter the estimation results, including the case when they mimic external stimuli, typical of task-based fMRI analysis. Finally, (c) if a misspecified Student-t spatial score-driven model is fitted to Gaussian data, then the average estimated degrees of freedom parameter results of the order of 105, with a median value $\hat{v}=\text{4, 834}$, in line with the results of Proposition 1.

## 4 Analysis of Resting State fmri Data

### 4.1 Data Description

The motivating dataset we analyze comes from a pilot study of the Enhanced Nathan Kline Institute-Rockland Sample project. This project aims at providing a large scale sample of publicly available multimodal neuroimaging data and psychological information to support researchers in the goal of understanding the mechanisms underlying the complex brain system; a detailed description of the project can be found at http://fcon_1000.projects.nitrc.org/indi/enhanced/. Our study comprises brain imaging data and subject-specific covariates for 16 subjects. Data are collected at 70 brain regions defined according to the anatomical parcellation based on the Desikan atlas (Desikan et al. 2006). For each region of interest (ROI), we have information on the 3D coordinates of its centroids, whether it belongs to the left or right hemisphere, and its anatomical lobe membership.

The dynamic functional activity is expressed as the noisy BOLD signal obtained during R-fMRI sessions. The data are recorded when the subject is not performing an explicit task during the imaging session while he/she is simply asked to stay awake with eyes open. The raw R-fMRI scans are preprocessed to derive the time series data for each brain region using the C-PAC software (https://fcp-indi.github.io/). Thus, for each subject, data are collected at 70 ROIs over 404 equally spaced time steps.

For each subject, the structural brain network made by white matter fibers connecting each pair of ROIs is also available. The white matter fibers are derived by diffusion tensor imaging (DTI) that maps the diffusion of water molecules across the biological brain tissues. The available structural network is obtained as the output of the pipeline ndmg described at http://m2g.io and consists of a 70 × 70 symmetric adjacency matrix measuring the total number of white matter fibers connecting each pair of brain regions in each subject. Such matrix is usually employed as an index of structural connectivity (Messé et al. 2015), that refers to how different brain regions are indeed physically connected.

Finally, personal information such as psychological disorder diagnosis, age, gender, and handedness are also collected for each subject. We take subject 6 and subject 13 as illustrative examples. Both subjects are under 25 years old and right-handed, but subject 6 suffers from major depressive disorder and abuse of cannabis, while subject 13 is healthy.

### 4.2 Weighted Anatomic Distance Matrix

A key ingredient required by our spatio-temporal model is the definition of a neighborhood structure among the brain regions. In the neuroimaging literature, specifying a spatial distance is not straightforward. Bowman (2007) discussed different measures of distance in the brain. One natural metric is the geometric or Euclidean distance, that measures the physical separation between two brain regions. Decreasing similarity between ROIs is expected with increasing geometric distance, that is, brain regions located close to each other tend to be functionally connected (Alexander-Bloch et al. 2013). However, as pointed out by Bowman (2007), the geometric distance fails to account for high correlations that may exist between far apart ROIs. Rather, long range interactions may depend on the number of structural connections of the brain regions (Tewarie et al. 2014). Thus, the second metric is provided by the anatomic distance, quantifying anatomic links connecting different brain locations. White matter bundles link cortical areas within the same hemisphere and areas in separate hemispheres, as well as areas in the cerebral cortex to various subcortical structures. Hence, white matter connections directly link brain structures that result to be anatomically close, even though geometrically distant.

In this work, the spatial neighborhood structure, assumed to be the same for the variables and the disturbances, that is, **W**_1_ = **W**_2_ = **W** (see Section 2), is built by taking into account both the geometric distance between pairs of ROIs and their anatomic distance based on white matter count. Specifically, let **D** be the symmetric matrix based on 3D Euclidean distance between the centroids of the ROIs, whose generic element is denoted as *d_i__j_*, for *i*, *j* = 1,…,*R*.Let F be the matrix containing the white matter fiber counts between ROIs *i* and *j* based on DTI, whose generic element is *f_i__j_*. We define spatial weights that are directly proportional to the number of white fibers *f_i__j_* and inversely proportional to the Euclidean distance *d_i__j_*, that is

(10) $$w_{ij}\propto \frac{f_{ij}d_{ij}^{-1}}{\sum_{i,j=1}^{R}f_{ij}d_{ij}^{-1}}.$$

Although **F** and **D** have different scales, the corresponding **W** is a scale free matrix by construction and as a by-product of the fact that it is row-stochastic.

**Figure 1:**
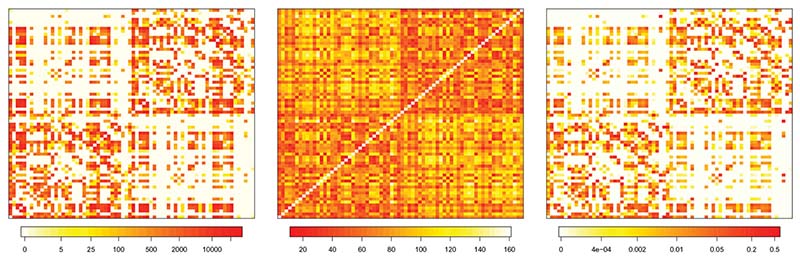
Left panel: representation of fiber count matrix (F) for subject 6. Middle panel: Euclidean distance among ROIs matrix (D), equal for all subjects. Right panel: spatial matrix (W) for subject 6 given as a weighted combination of F and D.

To visualize how the spatial weight matrix **W** in Equation (10) keeps into account the geometric and anatomic distance, Figure 1 reports the matrices **F**,**D**,**W** for one of the subjects analyzed, labeled as subject 6. The left panel reports the fiber count matrix **F**, the central panel shows the Euclidean distances matrix **D** while, in the right panel, the spatial matrix **W** is represented. We observe that the weight matrix is sparse. Such sparsity is mainly inherited from the white fiber matrix **F**. Indeed, for subject 6, we record 61% null connections, that is, white fiber count equal to zero.

We also note that higher weights are associated with ROIs belonging to the same hemisphere (the two main blocks of the matrix). However, since the matrix **F** is not symmetric with respect to the left and right hemisphere, the spatial matrix **W** may assign different weights to the same pair of regions located in opposite hemispheres; as an instance, for subject 6, the normalized weight associated with pair ROIs precentral and postcentral located in the left hemisphere is 0.116, while the weight corresponding to these two regions located in the right hemisphere is 0.102. Same considerations also hold for weights associated with cross-hemisphere regions.

Finally, for each subject, we employ an individual network of the entire brain, that is, the weight matrix **W** is subject-specific. This potentially allows us to compare individuals or groups, such as healthy subjects versus subjects with clinical conditions. For instance, we record different distributions of the non null connections between subjects 6 and 13, with mean white fiber count equal to 3375 (IQR 4101) and 2250.7 (IQR 3043.25), respectively. We account for such differences by the subject-specific matrix **W**.

### 4.3 Estimation Results

As a preliminary step, we tested the null hypothesis of multivariate normality on each subject. The results of the tests and additional exploratory analysis, reported and discussed in Section S8.1, lead us to conclude that the assumption of multinormality would not be appropriate for the data at hand.

Our analysis proceeds with the estimation of model parameters. We illustrate the results on subjects 6 and 13, for which the estimates of the spatial parameter *ρ*_2_ were significant at the level of 5% and equal to −0.1. A more parsimonious specification without the spatial autoregressive disturbance component has also been fitted (SAR score-driven model), which gave equivalent results in terms of estimated parameter values, likelihood and information criteria. For these reasons, the SAR score-driven specification has been preferred. The model comes with no fixed effects.

**Figure 2:**
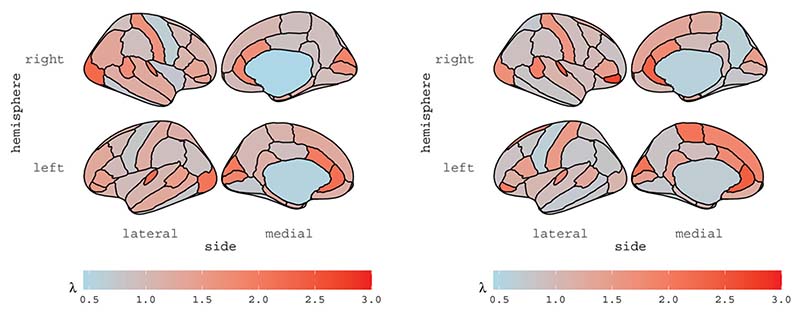
Estimates of vector exp{λ} for subject 6 (left panel) and subject 13 (right panel).

**Figure 3:**
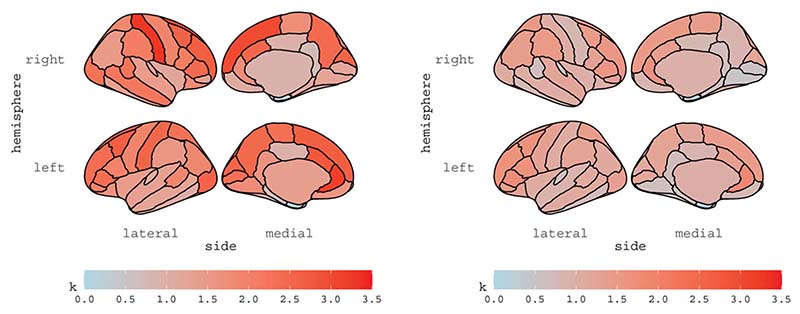
Estimates of vector *K* for subject 6 (left panel) and subject 13 (right panel).

The estimates of scalar parameters are reported in Table 1 for all subjects, with asymptotic standard errors computed based on the analytic conditional information matrix reported in Section S6. We note that all subjects show high temporal persistence, with all $\hat{\phi }$ s around 0.8, as well as high spatial dependence, with ${\hat{\rho }}_{1}$ s ranging from 0.66 to 0.84. The intercept $\hat{\beta_{0}}$ is basically zero for all subjects, as expected. In Section 2.2 we argued that the convergence of the multivariate Student-t distribution to the Normal distribution depends on the size of the multivariate space R; here, with *R* = 70, Table 1 confirms that a multivariate Normal assumption would be not appropriate for all subjects. The estimates of the vector parameters exp{λ} and K are reported for subjects 6 and 13 in Figure 2 and Figure 3, respectively; the estimates are displayed for each ROI, according to the Desikan atlas. The brain maps of the corresponding standard errors are reported in the Supplementary materials along with diagnostics checks and further discussions.

### 4.4 Detecting Spontaneous Activations

A procedure for detecting spontaneous activations is developed, based on the assumption that they correspond to extreme values of a possibly heavy-tailed distribution, as follows. Let ${\hat{Z}}_{1}{\hat{v}}_{t}$ be the spatial prediction error estimate from the SAR score-driven model and ${\hat{u}}_{t}$ be its winsorized version. If the data were actually generated by a Gaussian distribution, then **Z**_1_*_v_t__* = *u_t_* (both Gaussian), with *h_t_* = *Z_1v_t__*−*u_t_* being the null vector. One would then identify spontaneous activations like in Wang et al. (2008), as extreme quantiles of the Gaussian residuals ${\hat{Z}}_{1}{\hat{v}}_{t}$. If, on the other hand, the data are generated by a heavy-tailed Student-*t* distribution, then **Z**_1*v_t_*_ ≠ *u_t_* (heavy-tailed and thin-tailed, respectively, see Section 2) and *h_t_* = **Z**_1*vt*_ − *u_t_* = *b_t_Z_1_ v_t_*, where *b_t_* ∈ (0,1) is defined in Equation (9). Recall that ut is a winsorized version of *Z_1_v_t_*, tuned by the value of the degrees of freedom parameter (if υ → ∞ then *u_t_* = *Z_1_v_t_*). The diagonal matrix **K** further regulates the range of î *t* and, consequently, the distance between *Z_1_v_t_* and ut, as well as the robustness of the resulting filtered signal, yet ensuring that the each estimated residual ${\hat{Z}}_{1}{\hat{v}}_{t}$ is an upper bound for the corresponding scores $\hat{K}{\hat{u}}_{t}$.

Thus, the procedure for identifying brain activations in R-fMRI data rests on the comparison between the estimated residuals ${\hat{Z}}_{1}{\hat{v}}_{t}$ and $\hat{K}{\hat{u}}_{t}$. We choose to identify, as spontaneous activations, those values of ${\hat{Z}}_{1}{\hat{v}}_{t}$ that exceed the $\left(1-\frac{\alpha }{2R}\right)\%$ quantile of $\tilde{K}{\hat{u}}_{t}$, where ${\tilde{\kappa }}_{r}=\max\left\{{\hat{\kappa }}_{r},1\right\},r=1,\ldots ,R$, which reduces to the analysis of the quantile of ${\hat{Z}}_{1}{\hat{v}}_{t}$ in the Gaussian case. The Bonferroni correction is applied to control the falsepositive rate of *R* multiple comparisons at each time *t*. The conservative choice of $\left\{{\hat{\kappa }}_{r},1\right\}$ mitigates the effect of the degrees of freedom parameter; indeed, Figure 3 shows that the estimated values of κ for subject 6 (left panel) are much higher than those for subject 13 (right panel), opposite to the estimated degrees of freedom parameters, equal to $\hat{v}=36.49$ and $\hat{v}=\text{108}.71$, respectively. Note that, in principle, one can decide how to choose and correct the quantile of the robust or nonrobust residuals; the relevant point is to fit the most appropriate model to the data at hand.

**Figure 4:**
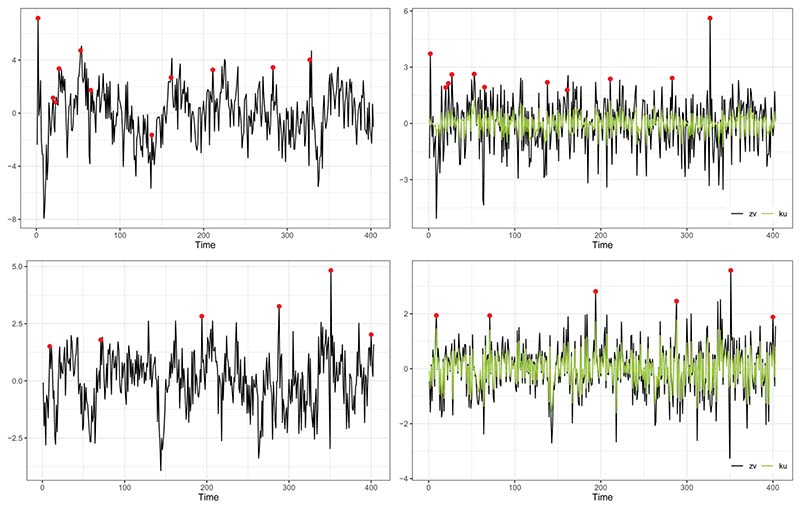
Left panel: R-fMRI times series and spontaneous activations (dots) detected in ROI lh-parahippocampal (left hemisphere, parahippocampal). Right panel: corresponding residuals and outliers (dots). Top panels: subject 6. Bottom panels: subject 13.

Figure 4 illustrates an example of spontaneous activations detected for ROI lh-parahippocampal for the two subjects at α = 0.05. The left panels show the original R-fMRI time series with potential anomalies highlighted with dots. Such values are identified as the residuals ${\hat{Z}}_{1}{\hat{v}}_{t}$, displayed in the right panels, that exceed the quantile-based threshold. The figure shows how the strategy for detecting spontaneous activations based on the residuals of the fitted model applies in practice: the detected anomalies could not have been identified from the raw time series. In the top row of the figure, we display subject 6, while in the bottom we focus on subject 13. We note that, at the fixed ROI, the estimated spatial prediction error ${\hat{Z}}_{1}{\hat{v}}_{t}$ (black line) and its winsorized version $\hat{K}{\hat{u}}_{t}$ (green line) are closer in subject 13 than in subject 6. Indeed, we recall that the estimates of v are 36.49 and 108.71 for subjects 6 and 13, respectively (see Section 4.3).

**Figure 5:**
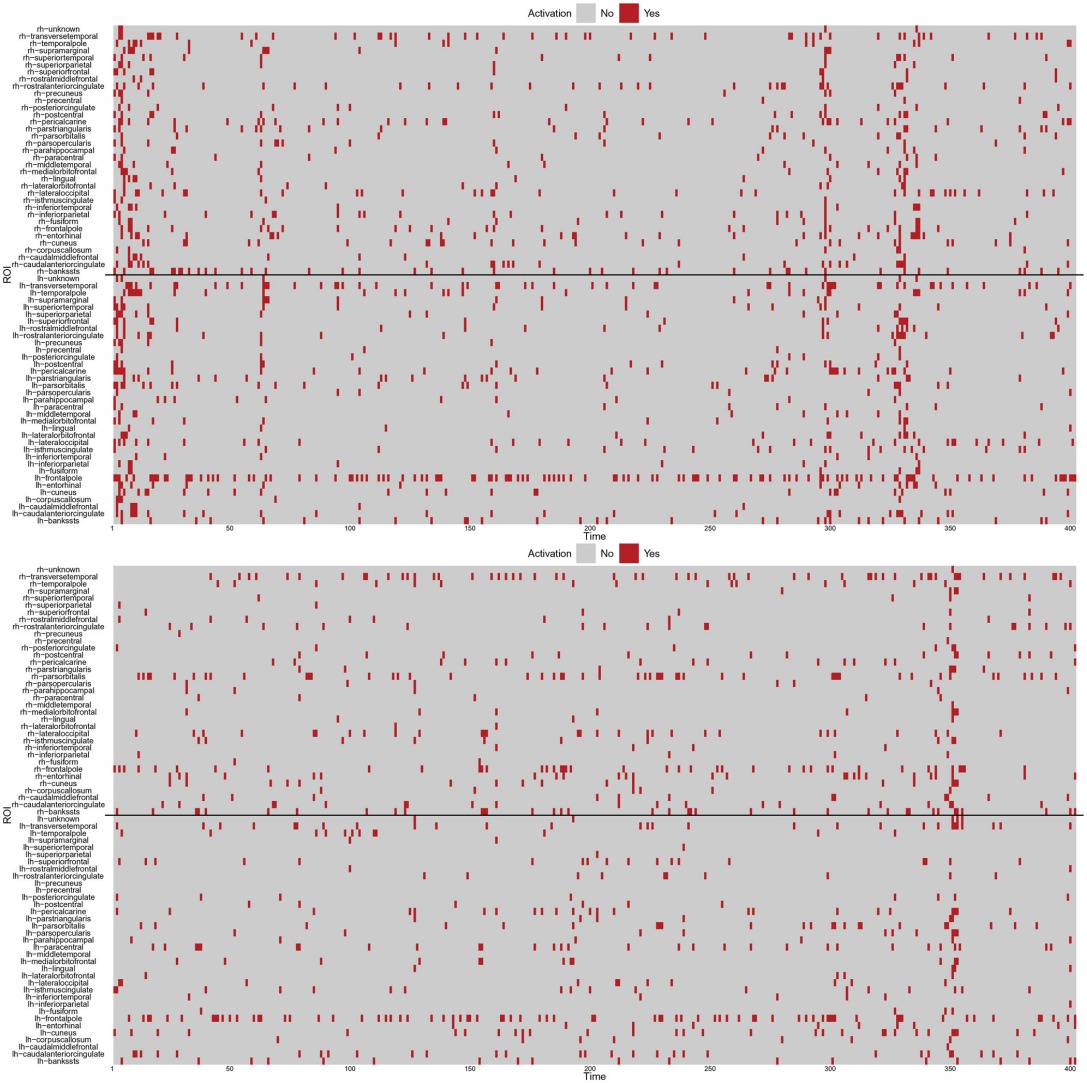
Spontaneous activations detected for subject 6 (top) and 13 (bottom) over time (x-axis) for all ROIs (y-axis); above the black line we record the ROI in the right hemisphere, while below the black line we record the ROIs in the left hemisphere.

Spontaneous activations detected for all ROIs are summarized in Figure 5 for subjects 6 (top panel) and 13 (bottom panel). The solid black lines distinguish the ROIs of the left and right hemispheres, that is, spontaneous activations of ROIs in the right hemisphere are above the line, while the ones in the left hemisphere are below the line. Overall, we see that less activations are recorded for the healthy subject 13. Moreover, the figure shows that, at given time steps, spontaneous activations are detected for most of the ROIs (see for instance at time 350 in the bottom panel). With a different viewpoint, we note that few ROIs keep being activated during the observational time frame (see for example ROI lh-frontalpole). In order to facilitate the interpretation, videos that provide a dynamic spatial view of the activations are included as supplementary materials.

**Figure 6:**
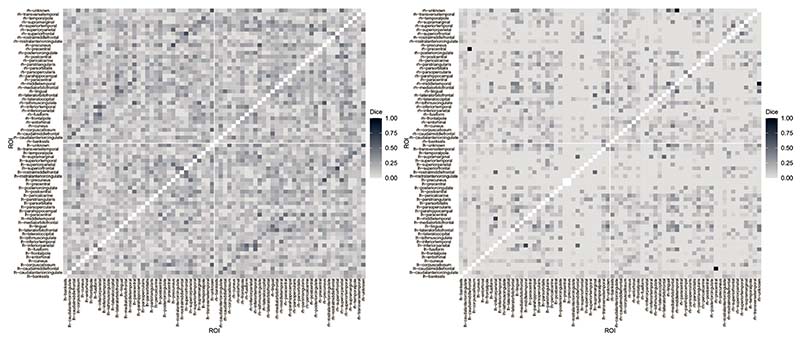
Dice similarity index computed across activations recorded over time for subject 6 (left panel) and subject 13 (right panel).

To conclude, we compute the Dice index (Dice 1945) among the detected spontaneous activations. The index is a measure of similarity between binary elements, that is, it provides an empirical measure of spontaneous co-activations between ROIs (Liu et al. 2018). The index ranges between 0 and 1; the higher the index between two activations series (two rows of the matrices represented in Figure 5), the more similar the two ROIs with respect to the spontaneous activations are. We report the Dice index computed for subjects 6 and 13 in Figure 6. We observe that the index is low in general for both subjects, as it is on average 0.14 (sd 0.10) for subject 6 and 0.05 (sd 0.09) for subject 13. The computation of the Dice index was also exploited by Gasperoni and Luati (2018) after having fitted a robust time series model, independently at each ROI. It is interesting to highlight that the average Dice index for a specific subject is slightly lower in the spatio-temporal framework with respect to the univariate setting (mean Dice index equal to 0.16 (sd 0.12) for subject 6 and 0.07 (sd 0.12) for subject 13). This change is expected since the current model adjusts for the spatial structure of BOLD signals. Figure 6 also reveals that higher values of the index are associated with the same pair of ROIs belonging to opposite hemispheres, represented by the darker diagonals in the top-left and bottom-right blocks of the matrices.

## 5 Discussion

A robust spatio-temporal model has been developed to analyze areal data collected over time and coming from a possibly heavytailed distribution. The basic assumption was that of a conditional Student-t distribution for the data generating process, that relaxed the widely adopted, yet questioned, assumption of normality. One point of strength of the model consists in its flexibility, as the multivariate normal distribution is encompassed as a special case when the degrees of freedom parameter tends to infinity. We observed that convergence to the multivariate normal distribution may become slower as long as the number of analyzed time series increases. In practice, for R = 70, fitting a misspecified Student-t model on Gaussian data resulted in an average value of the estimated degrees of freedom parameter of the order of 105 (median nearly at R2), thus dispelling the possibility of carrying over misspecified inference based on the Student-t spatial score-driven model.

On a theoretical viewpoint, the stochastic properties of the model, such as stationarity and invertibility, and the inferential properties of maximum likelihood estimators, that is, consistency and asymptotic normality, have been derived, for a fixed spatial and a diverging temporal dimension. The analytic expression of the conditional information matrix is also provided. The main technical difficulties, also in view of possible extensions to spatial asymptotic theory, as in Yu, de Jong, and Lee (2008), Lee and Yu (2010) and Robinson and Rossi (2015), are related to the nonlinearity of the model, coming from the score, and eventually implying the robustness of the resulting filter.

On the applied side, besides going beyond the normality assumption, the novelty brought by the multimodal model in the R-fMRI literature concerned (i) the construction of a spatial weight matrix based on the combination of different metrics and (ii) the detection of spontaneous activations based on the residuals of a robust signal extraction. As far as (i) is concerned, by including information from DTI data (white fiber counts) in the definition of the weight matrix has allowed us to exploit a subject-specific measure of structural connectivity. More sophisticated measures of structural connectivity can be further exploited, for instance by accounting for geometric features of fiber curves (Zhang, Descoteaux, and Dunson 2019). Regarding (ii), the flexible model-based approach developed in the article opens the way to a different paradigm for detecting spontaneous activations in R-fMRI data. In addition, the inclusion of explanatory variables makes the model fully applicable also to fMRI data recorded in task-based experiments.

## Supplementary Material

Code & Data

Code contents

Supplementary Material

Video 1

Video 2

## Figures and Tables

**Table 1:** Estimates of the scalar static parameters of the model for all subjects. Asymptotic standard errors are reported in brackets.

	subjects															
	1	2	3	4	5	6	7	8	9	10	11	12	13	14	15	16
$\hat{\phi }$	0.815 (0.006)	0.701 (0.006)	0.699 (0.008)	0.738 (0.005)	0.810 (0.004)	0.828 (0.004)	0.789 (0.005)	0.799 (0.004)	0.787 (0.004)	0.743 (0.006)	0.852 (0.003)	0.709 (0.005)	0.803 (0.004)	0.849 (0.006)	0.650 (0.006)	0.786 (0.004)
${\hat{\rho }}_{1}$	0.710 (0.009)	0.753 (0.004)	0.658 (0.006)	0.784 (0.007)	0.765 (0.003)	0.731 (0.003)	0.797 (0.004)	0.772 (0.004)	0.779 (0.005)	0.783 (0.005)	0.826 (0.005)	0.772 (0.003)	0.661 (0.004)	0.672 (0.008)	0.785 (0.003)	0.784 (0.004)
${\hat{\beta }}_{0}$	0.006 (0.110)	0.006 (0.067)	0.004 (0.075)	0.023 (0.089)	0.017 (0.114)	−0.013 (0.116)	0.011 (0.101)	0.013 (0.110)	−0.002 (0.104)	0.013 (0.101)	−0.054 (0.170)	0.012 (0.117)	−0.002 (0.154)	0.009 (0.094)	−0.001 (0.108)	0.009 (0.111)
$\hat{v}$	82.216 (17.650)	142.204 (14.940)	292.837 (69.336)	41.782 (6.888)	69.645 (6.668)	36.485 (3.081)	149.722 (16.234)	60.648 (4.533)	56.911 (5.647)	62.087 (7.394)	15.874 (2.196)	93.069 1 (6.760)	08.711 (8.565)	176.853 (49.160)	195.265 (18.418)	46.044 (4.994)
