# Supplementary material for: Score-Driven Modeling of Spatio-Temporal Data
Source: J Am Stat Assoc. 2021 Oct 4;118(542):1066–77. doi: 10.1080/01621459.2021.1970571 (PMC7614622; doi:10.1080/01621459.2021.1970571)
Supplement: Supplemental Material [file UASA_A_1970571_SM6649.zip › GLPD_supplementary_materials.zip!/code&data/readme.pdf]

# DATA

The dataset comes from a pilot study of the Enhanced Nathan Kline Institute-Rockland Sample project. A detailed description of the project, scopes, and technical aspects can be found at http://fcon_1000.projects.nitrc.org/indi/enhanced/.

The raw data have been pre-processed by Greg Kiar and Eric Bridgeford (NeuroData, Johns Hopkins University) who made the data available to the participants of StartUp research meeting, held on June 25-27, 2017 (http://www.congressi.unisi.it/startupresearch/).

The dataset is contained in the "code&data.zip" as follows:

- the file *data_ts_complete.RData* contains the BOLD signals collected at 70 ROIs over 404 equally spaced time steps for the 16 subjects [R-fMRI, BOLD data for 16 subjects, 70X404X16];
- the file *fibre_count.RData* contains the DTI data, i.e., the 70 x 70 symmetric adjacency matrix measuring the total number of white matter fibers connecting each pair of brain regions in each subject [DTI data for 16 subjects, 70X70X16];
- the file *subj_cov_paper.RData* contains the covariate information for each subject [subject covariates, 16X7];
- the file *ROI-covariates.txt* contains the information on the 70 ROIs [ROI characteristics, 70X6].

# CODE

We provide the code to reproduce the main results shown in the manuscript: (i) Maximum Likelihood Estimators; (ii) Standard Errors computation according to the asymptotic theory provided in the supplementary material; (iii) spontaneous activation detection; (iv) empirical coverage estimate. It is possible to run the code on the data associated to the real case study.

Main script in which we perform the maximum likelihood estimates, the standard errors computation and the spontaneous activation detection:

- main.R

Likelihood function to be maximized:

- SARAR_general_filter.R

Functions required to compute the standard errors:

- mDCS_t_Model_v2.R
- mLibraries_Utilities.R
- mStudent_t.R
- mDCS_t_Scores.R
- mDCS_t_Cond_Informations_v4.R
- mDCS_t_Fisher_Scoring.R
- mStarting_Parameters.R

Residuals computation:

- SARAR_general_eval.R

Empirical coverage estimate:

- Confidence_Bands_Filter.R
