# Supplementary material for: Score-Driven Modeling of Spatio-Temporal Data
Source: J Am Stat Assoc. Author manuscript; Available in PMC 2023 Jun 5. (PMC7614622; doi:10.1080/01621459.2021.1970571)
Supplement: Supplementary file 1 [file EMS140627-supplement-Supplementary_Material.pdf]

Supplementary materials for: 'Score driven modeling of spatio-temporal data'

Francesca Gasperoni[*], Alessandra Luati[†], Lucia Paci[‡], Enzo D'Innocenzo[†]

Introduction

This document contains supplementary materials to the main manuscript. In particular, we provide and discuss here the proof of Propositions 1, 2 and 3 as well as the proof of Theorems 3.1 and 3.2. In Section S6 we derive the conditional information matrix complementing Theorem 3.2. Section S7 presents the results of an extensive simulation study carried out to assess the finite sample properties of the maximum likelihood estimator, the impact of different kind of exogenous variables and the effects of potential misspecification. Finally, in Section S8, we display additional empirical results, including preliminary analysis on R-fMRI data and diagnostic checks.

S1 Proof of Proposition 1

Let us write the R-variate Student-t density with zero mean, unit scale and ν degrees of freedom as

$$f(\boldsymbol{y}_t) = (2\pi)^{-\frac{R}{2}} k_\nu(\boldsymbol{y}_t) c_\nu \tag{S1}$$

where

$$k_\nu(\boldsymbol{y}_t) = \left(1 + \frac{\boldsymbol{y}_t' \boldsymbol{y}_t}{\nu}\right)^{-\frac{\nu+R}{2}}$$

[*]MRC Biostatistics Unit, University of Cambridge
[†]Department of Statistical Sciences, University of Bologna
[‡]Department of Statistical Sciences, Università Cattolica del Sacro Cuore

and

$$c_\nu = \frac{\Gamma\left(\frac{\nu}{2}+\frac{R}{2}\right)}{\Gamma\left(\frac{\nu}{2}\right)\left(\frac{\nu}{2}\right)^{\frac{R}{2}}}.$$

It is straightforward to prove (see Exercise 4.36 of Abadir et al. 2018) that

$$\lim_{\nu\to\infty} k_\nu(\boldsymbol{y}_t) = \exp\left\{-\frac{\boldsymbol{y}_t'\boldsymbol{y}_t}{2}\right\}$$

and

$$\lim_{\nu\to\infty} c_\nu = 1$$

so that

$$\lim_{\nu\to\infty} f(\boldsymbol{y}_t) = \phi(\boldsymbol{y}_t)$$

where $\phi(\boldsymbol{y}_t)$ denotes the multivariate standard normal density.

Let us consider the following expansion of $k_\nu(\boldsymbol{y}_t)$ and, subsequently, an asymptotic expansion of c_ν, valid for $\nu\to\infty$.

We first rewrite $k_\nu(\boldsymbol{y}_t)$ and expand the logarithmic term, as follows,

$$\begin{aligned} k_\nu(\boldsymbol{y}_t) &= \exp\left\{-\tfrac{\nu+R}{2}\log\left(1+\tfrac{\boldsymbol{y}_t'\boldsymbol{y}_t}{\nu}\right)\right\} \\ &= \exp\left\{-\tfrac{\nu+R}{2}\left(\tfrac{\boldsymbol{y}_t'\boldsymbol{y}_t}{\nu}-\tfrac{1}{2}\tfrac{(\boldsymbol{y}_t'\boldsymbol{y}_t)^2}{\nu^2}+O\left(\tfrac{1}{\nu^3}\right)\right)\right\}. \end{aligned}$$

Rearranging,

$$\begin{aligned} k_\nu(\boldsymbol{y}_t) &= \exp\left\{-\tfrac{\nu+R}{\nu}\left(\tfrac{\boldsymbol{y}_t'\boldsymbol{y}_t}{2}-\tfrac{1}{2}\tfrac{(\boldsymbol{y}_t'\boldsymbol{y}_t)^2}{2\nu}+O\left(\tfrac{1}{\nu^2}\right)\right)\right\} \\ &= \exp\left\{-\tfrac{\boldsymbol{y}_t'\boldsymbol{y}_t}{2}+\tfrac{(\boldsymbol{y}_t'\boldsymbol{y}_t)^2-2R\boldsymbol{y}_t'\boldsymbol{y}_t}{4\nu}+O\left(\tfrac{1}{\nu^2}\right)\right\} \\ &= \exp\left\{-\tfrac{\boldsymbol{y}_t'\boldsymbol{y}_t}{2}\right\}\exp\left\{\tfrac{(\boldsymbol{y}_t'\boldsymbol{y}_t)^2-2R\boldsymbol{y}_t'\boldsymbol{y}_t}{4\nu}+O\left(\tfrac{1}{\nu^2}\right)\right\}. \end{aligned}$$

By expanding $\exp\{x\} = 1 + x + O(x^2)$ when $x\to 0$, we eventually get

$$k_\nu(\boldsymbol{y}_t) = \exp\left\{-\frac{\boldsymbol{y}_t'\boldsymbol{y}_t}{2}\right\}\left(1+\frac{(\boldsymbol{y}_t'\boldsymbol{y}_t)^2-2R\boldsymbol{y}_t'\boldsymbol{y}_t}{4\nu}+O\left(\frac{1}{\nu^2}\right)\right). \tag{S2}$$

As far as c_ν is concerned, we consider the Stirling formula (see Appendix A.3.4 of Abadir et al. 2018), based of Tricomi and Erdelyi (1951),

$$c_\nu = 1+\frac{R\,(R-2)}{4\nu}+O\left(\frac{1}{\nu^2}\right). \tag{S3}$$

By replacing (S2) and (S3) in (S1) and solving the product, one has

$$f(\boldsymbol{y}_t) = (2\pi)^{-\frac{R}{2}} \exp\left\{-\frac{\boldsymbol{y}_t'\boldsymbol{y}_t}{2}\right\} \left(1 + \frac{R^2 - 2R(1+\boldsymbol{y}_t'\boldsymbol{y}_t) + (\boldsymbol{y}_t'\boldsymbol{y}_t)^2}{4\nu} + O\left(\frac{1}{\nu^2}\right)\right)$$

which completes the proof. □

S1.1 Discussion

It is evident from Equation (S3) that the term involving R^2/ν comes from the integrating constant. To illustrate, the left panel of Figure S1 shows how fast the constant c_ν departs from unity as far as R increases and $\nu \in [30, 80]$. It is evident that, already for $R = 10$, c_ν is far from 1 even for $\nu = 80$. The right panel of Figure S1 compares the behaviour of c_ν for fixed $\nu = 30R$ (orange line) and fixed $\nu = 30R^2$ (blu curve). It emerges that values of $\nu > 30R^2$ are required for c_ν to stably reach unity, i.e. its limit. It is worth noting that the two curves overlap for small values of R. The value $\nu_0 = 30R^2$ encompasses the case when $R = 1$ and $\nu_0 = 30$ emerging from Fisher (1925) and established by Chu (1956). The latter value can be interpreted as a turning point, in the terminology of Villa and Walker (2014), after which the Student-t distribution can be reliably approximated by the Normal. Note, however, that in practical applications the kernel plays a role as well.

Indeed, the kernel expansion in Equation (S2) entails a more involved relation between R and quadratic forms associated with $\boldsymbol{y_t}$. The latter relation is discussed by Tiao and Zellner (1964), in the context of Bayesian estimation in regression models, and by Dickey (1967) who considered alternative expansions, to the one in Fisher (1925), of the Student-t density function. Dickey (1967) also mentions the extension to the multivariate Student-t distribution and recognises that, in the multivariate case, expansions of complete integrals for termwise integration, as in the univariate case treated by Fisher (1925), are practically rather complicate.

In Proposition 1, we recover the leading terms of the expansions considered in Tiao and Zellner (1964) and Dickey (1967). As a matter of fact, the term in ν^{-1} in Equation (S2) is equivalent to the term in ν^{-1} in the multivariate Student-t kernel expansion of Tiao and Zellner (1964, Equation 3.2). Also, writing $k_\nu(\boldsymbol{y}_t)$ as follows,

$$k_\nu(\boldsymbol{y}_t) = \exp\left\{-\frac{\boldsymbol{y}_t'\boldsymbol{y}_t}{2}\frac{\nu+R}{\nu}\left(1 - \frac{\boldsymbol{y}_t'\boldsymbol{y}_t}{2\nu} + O\left(\frac{1}{\nu^2}\right)\right)\right\},$$

gives the first terms of the kernel density expansions in Appell's polynomials discussed by Dickey (1967), see also Kotz and Nadarajah (2004, Section 1.6).

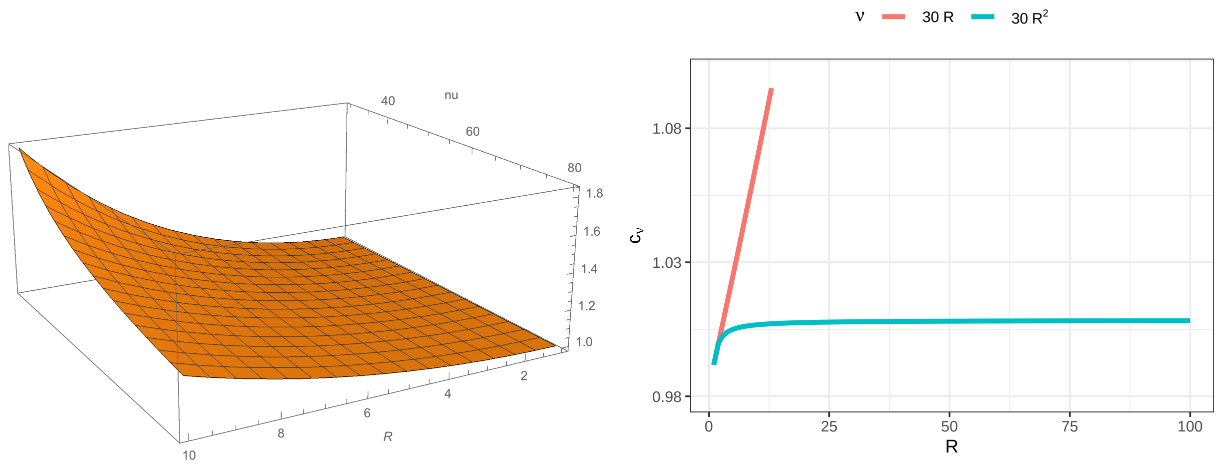

Figure S1: Left panel: values of c_ν for $R = 1, 2, \dots, 10$ and $\nu = 30, \dots, 80$. It is evident that, already for $R = 10$, c_ν is far from 1 even for $\nu = 80$. Right panel: values of c_ν for $R = 1, 2, \dots, 100$ and fixed $\nu = 30R$ (orange line) and fixed $\nu = 30R^2$ (blu curve). One can see that c_ν remains stable around one for $\nu_0 = 30R^2$ while increases linearly for $\nu = 30R$.

S2 Proof of Proposition 2

We first prove stationarity and ergodicity of $\{\boldsymbol{y}_t\}_{t\in\mathbb{Z}}$ in Equation (1), which requires stationarity and ergodicity of $\{\boldsymbol{\mu}_t\}_{t\in\mathbb{Z}}$ along with some boundedness conditions on the spatial matrices $\mathbf{W}_1$ and $\mathbf{W}_2$ and coefficients ρ_1 and ρ_2. The sequence of $\boldsymbol{\varepsilon}_t$ is iid, and hence, stationary and ergodic by definition. For $0 < \nu < \infty$, the random variable $1/(1+\boldsymbol{\eta}_t^\top \boldsymbol{\Lambda}^{-1}\boldsymbol{\eta}_t/\nu)$ is distributed like a Beta$\,(R/2, \nu/2)$, and hence is uniformly bounded $\forall \boldsymbol{\eta}_t \in \mathbb{R}^R$, see Kotz and Nadarajah (2004, pp. 19) and Harvey (2013, Proposition 39). As, for given $\boldsymbol{\eta}_t$, the recursion that specifies $\boldsymbol{\mu}_{t+1}$ in Proposition 2 is linear in $\boldsymbol{\mu}_t$, with the assumptions $|\phi| < 1$, the process $\{\boldsymbol{\mu}_t\}_{t\in\mathbb{Z}}$ is stationary and ergodic, since all the conditions of Bougerol (1993, Theorem 3.1) are satisfied.

From the fact that $|\rho_1| < 1$, $|\rho_2| < 1$, and that $\mathbf{W}_1$, $\mathbf{W}_2$ are row-stochastic, which in turn imply $||\rho_1 \mathbf{W}_1|| < 1$ and $||\rho_2 \mathbf{W}_2|| < 1$, two constants $0 < c_1, c_2 < 1$ exist, such that for $i = 1, 2$

$$\left\| \sum_{m=1}^{\infty} (\rho_i \mathbf{W}_i)^m \right\| \leq \sum_{m=1}^{\infty} \|\rho_i \mathbf{W}_i\|^m = \sum_{m=1}^{\infty} c_i^m = \frac{1}{1 - c_i} < \infty.$$

As $\mathbf{X}_t$ is non stochastic and bounded by assumption, $\{\boldsymbol{\mu}_t\}_{t\in\mathbb{Z}}$ is stationary and ergodic,

and $\{\boldsymbol{\eta}_t\}_{t\in\mathbb{Z}}$ is iid, stationarity of $\{\boldsymbol{y}_t\}_{t\in\mathbb{Z}}$ then follows trivially by continuity arguments, whereas ergodicity is implied by Krengel and Brunel (1985, Proposition 4.3).

Turning to boundedness of the unconditional moments of $\boldsymbol{y}_t$, we observe that $\mathbb{E}[\|\boldsymbol{\mu}_t\|^n] < \infty$ follows by an application of the Hölder and Minkowski's inequality, since

$$\mathbb{E}\left[\|\boldsymbol{\mu}_{t+1}\|^n\right] \leq \left\{ \bar{\kappa} \sum_{j=0}^{\infty} \phi^j \left(\mathbb{E}\left[\left\| \frac{\boldsymbol{\eta}_{t-j}}{1+\boldsymbol{\eta}_{t-j}^\top \boldsymbol{\Lambda}^{-1} \boldsymbol{\eta}_{t-j}/\nu} \right\|^n \right] \right)^{1/n} \right\}^n < \infty,$$

where $\bar{\kappa} = R\|\mathbf{K}\|$ and $\phi < 1$, by boundedness of $\mathbf{W}_1$ and $\mathbf{W}_2$ (by construction) and by positive definiteness of $\mathbf{K}$ (by assumption).

Finally, by the c_r-inequality, $\exists\, C_1, C_2 \in (0,\infty)$ such that

$$\mathbb{E}[\|\boldsymbol{y}_t\|^n] \leq \frac{C_1}{1-c_1}\|\mathbf{X}_t\boldsymbol{\beta}\|^n + \frac{C_1}{1-c_1}\mathbb{E}[\|\boldsymbol{\mu}_t\|^n] + \frac{C_1}{1-c_1}\frac{C_2}{1-c_2}\mathbb{E}[\|\boldsymbol{\eta}_t\|^n] < \infty,$$

since $\boldsymbol{\mu}_t$ is uniformly bounded, $\|\mathbf{X}_t\|^n < \infty$ with $\lim_{T\to\infty} \frac{1}{T}\mathbf{X}_t^T\mathbf{X}_t$ by assumption, and $\mathbb{E}\left[\|\boldsymbol{\eta}_t\|^n\right] < \infty$ for $n \geq \nu$ by the properties of the Student-t distribution. □

S3 Proof of Proposition 3

The proof consists in verifying that the sufficient conditions for invertibility given by Bougerol (1993, Theorem 3.1) are satisfied. As far as condition C.1 of Bougerol (1993, Theorem 3.1) is concerned, under the assumptions of Proposition 2, the filter $\boldsymbol{\mu}_{t|t-1}$ is uniformly bounded and hence $\mathbb{E}(\sup_{\boldsymbol{\theta}\in\boldsymbol{\Theta}} \|\boldsymbol{\mu}_{t|t-1}\|) < \infty$. Condition C.2 is satisfied by assuming the contraction assumption on $\boldsymbol{\mathcal{X}}_t$. Specifically, the convergence of the nonstationary filtered sequence $\{\hat{\boldsymbol{\mu}}_{t|t-1}\}_{t\in\mathbb{N}}$ is obtained as an application of the mean value theorem, so that

$$\sup_{\boldsymbol{\theta}\in\boldsymbol{\Theta}} \|\hat{\boldsymbol{\mu}}_{t+1|t} - \boldsymbol{\mu}_{t+1|t}\| \leq \sup_{\boldsymbol{\theta}\in\boldsymbol{\Theta}} \|\boldsymbol{\mathcal{X}}_t\| \sup_{\boldsymbol{\theta}\in\boldsymbol{\Theta}} \|\hat{\boldsymbol{\mu}}_{t|t-1} - \boldsymbol{\mu}_{t|t-1}\|,$$

with $\boldsymbol{\mathcal{X}}_t = \phi \boldsymbol{I}_R + \mathbf{K}\boldsymbol{\mathcal{C}}_t$ and where

$$\boldsymbol{\mathcal{C}}_t = \frac{\partial \boldsymbol{u}_t}{\partial \boldsymbol{\mu}_{t|t-1}^\top} = 2/\nu\alpha_t^{-2}\left(\mathbf{Z}_1\boldsymbol{y}_t - \mathbf{X}_t\boldsymbol{\beta} - \boldsymbol{\mu}_{t|t-1}\right)\left(\mathbf{Z}_1\boldsymbol{y}_t - \mathbf{X}_t\boldsymbol{\beta} - \boldsymbol{\mu}_{t|t-1}\right)^\top \mathbf{Z}_2^\top \boldsymbol{\Lambda}^{-1}\mathbf{Z}_2 - \alpha_t^{-1}\boldsymbol{I}_R,$$

is uniformly bounded $\forall \boldsymbol{y}_t \in \mathbb{R}^R$, $\forall\, \mathbf{X}_t \in \mathbb{R}^{R\times(p+1)}$, and $\forall \boldsymbol{\theta} \in \boldsymbol{\Theta}$. The contraction condition ensures that $\sup_{\boldsymbol{\theta}\in\boldsymbol{\Theta}} \|\hat{\boldsymbol{\mu}}_{t|t-1} - \boldsymbol{\mu}_{t|t-1}\| \xrightarrow{\text{e.a.s.}} 0$. Additionally, the claim that the moments are bounded follow from uniform boundedness of $\boldsymbol{u}_t$, see Proposition 2.

S4 Proof of Theorem 3.1

Let $\mathcal{L}_T(\boldsymbol{\theta}) = \frac{1}{T}\sum_{t=1}^{T}\ell_t(\boldsymbol{\theta})$ be the average likelihood, with limit $\mathcal{L}(\boldsymbol{\theta}) = \mathbb{E}[\ell_t(\boldsymbol{\theta})]$ and $\hat{\mathcal{L}}_T(\boldsymbol{\theta}) = \frac{1}{T}\sum_{t=1}^{T}\hat{\ell}_t(\boldsymbol{\theta})$ be the empirical average likelihood based on the recursion $\hat{\boldsymbol{\mu}}_{t|t-1}$ corresponding to the fixed initial value $\boldsymbol{\mu}_{1|0}$. We have,

$$\sup_{\boldsymbol{\theta}\in\boldsymbol{\Theta}}\left|\widehat{\mathcal{L}}_T(\boldsymbol{\theta}) - \mathcal{L}(\boldsymbol{\theta})\right| \le \sup_{\boldsymbol{\theta}\in\boldsymbol{\Theta}}\left|\widehat{\mathcal{L}}_T(\boldsymbol{\theta}) - \mathcal{L}_T(\boldsymbol{\theta})\right| + \sup_{\boldsymbol{\theta}\in\boldsymbol{\Theta}}\left|\mathcal{L}_T(\boldsymbol{\theta}) - \mathcal{L}(\boldsymbol{\theta})\right|. \tag{S4}$$

Let us consider the first term on the right hand side of Equation (S4). By the mean value theorem,

$$\sup_{\boldsymbol{\theta}\in\boldsymbol{\Theta}}\left|\widehat{\mathcal{L}}_T(\boldsymbol{\theta}) - \mathcal{L}_T(\boldsymbol{\theta})\right| \le \sup_{\boldsymbol{\theta}\in\boldsymbol{\Theta}}\left\|\frac{\partial\widehat{\mathcal{L}}_T(\boldsymbol{\theta})}{\partial\hat{\boldsymbol{\mu}}^{\star\top}_{t|t-1}}\right\| \sup_{\boldsymbol{\theta}\in\boldsymbol{\Theta}}\left\|\hat{\boldsymbol{\mu}}_{t|t-1} - \boldsymbol{\mu}_{t|t-1}\right\|, \tag{S5}$$

where $\hat{\boldsymbol{\mu}}^{\star}_{t|t-1}$ lies on the chord between the filtered location $\hat{\boldsymbol{\mu}}_{t|t-1}$ and $\boldsymbol{\mu}_{t|t-1}$. By direct calculation and the triangle inequality,

$$\begin{aligned}
\sup_{\boldsymbol{\theta}\in\boldsymbol{\Theta}}\left\|\frac{\partial\widehat{\mathcal{L}}_T(\boldsymbol{\theta})}{\partial\hat{\boldsymbol{\mu}}^{\star\top}_{t|t-1}}\right\| \le& \frac{1}{T}\sum_{t=1}^{T}\sup_{\boldsymbol{\theta}\in\boldsymbol{\Theta}}\left\|\frac{\nu+R}{\nu}\mathbf{Z}_2^\top\boldsymbol{\Lambda}^{-1}\mathbf{Z}_2(\mathbf{Z}_1\boldsymbol{y}_t - \mathbf{X}_t\boldsymbol{\beta} - \hat{\boldsymbol{\mu}}^{\star}_{t|t-1})/\alpha^\star_t\right\| \\
\le& c_{\boldsymbol{\Lambda}}\left(\max_{\boldsymbol{\theta}\in\boldsymbol{\Theta}}\frac{\nu+R}{\nu}\right)\frac{1}{T}\sum_{t=1}^{T}\sup_{\boldsymbol{\theta}\in\boldsymbol{\Theta}}\left\|(\mathbf{Z}_1\boldsymbol{y}_t - \mathbf{X}_t\boldsymbol{\beta} - \hat{\boldsymbol{\mu}}^{\star}_{t|t-1})/\alpha^\star_t\right\|,
\end{aligned}$$

with $c_{\boldsymbol{\Lambda}} > 0$ and $\alpha^\star_t = 1 + (1/\nu)\left(\mathbf{Z}_1\boldsymbol{y}_t - \mathbf{X}_t\boldsymbol{\beta} - \hat{\boldsymbol{\mu}}^{\star}_{t|t-1}\right)^\top\mathbf{Z}_2^\top\boldsymbol{\Lambda}^{-1}\mathbf{Z}_2\left(\mathbf{Z}_1\boldsymbol{y}_t - \mathbf{X}_t\boldsymbol{\beta} - \hat{\boldsymbol{\mu}}^{\star}_{t|t-1}\right)$. Note that $\|(\mathbf{Z}_1\boldsymbol{y}_t - \mathbf{X}_t\boldsymbol{\beta} - \hat{\boldsymbol{\mu}}^{\star}_{t|t-1})/\alpha^\star_t\|$ is uniformly bounded $\forall\boldsymbol{y}_t \in \mathbb{R}^R$, $\forall\mathbf{X}_t \in \mathbb{R}^{R\times(p+1)}$, and $\forall\boldsymbol{\theta} \in \boldsymbol{\Theta}$. The second term in the right hand side of Equation (S5) will vanish as $t \to \infty$ by Proposition 3. Almost sure convergence of the first term in the right hand side of Equation (S4) is then obtained as a corollary of Straumann and Mikosch (2006, Lemma 2.1).

As far as the second term in the right hand side of (S4) is concerned, convergence is obtained as an application of the uniform law of large numbers for stationary and ergodic processes, see White (1994, Theorem A.2.2). First, stationarity and ergodicity of $\{\ell_t(\boldsymbol{\theta})\}_{t\in\mathbb{Z}}$ follows by Propositions 2 and 3.

Second, we show that $\mathbb{E}(\sup_{\boldsymbol{\theta}\in\boldsymbol{\Theta}} |\ell_t(\boldsymbol{\theta})|) < \infty$. We have

$$\mathbb{E}\left[\sup_{\boldsymbol{\theta}\in\boldsymbol{\Theta}} |\ell_t(\boldsymbol{\theta})|\right] \leq \sup_{\boldsymbol{\theta}\in\boldsymbol{\Theta}}\left|\ln\Gamma\left(\frac{\nu+R}{2}\right)\right| + \sup_{\boldsymbol{\theta}\in\boldsymbol{\Theta}}\left|\ln\Gamma\left(\frac{\nu}{2}\right)\right| + \sup_{\boldsymbol{\theta}\in\boldsymbol{\Theta}}\left|\frac{R}{2}\ln(\pi\nu)\right|$$
$$+ \sup_{\boldsymbol{\theta}\in\boldsymbol{\Theta}}\big|\ln|\mathbf{Z}_1|\big| + \sup_{\boldsymbol{\theta}\in\boldsymbol{\Theta}}\big|\ln|\mathbf{Z}_2|\big| + \sup_{\boldsymbol{\theta}\in\boldsymbol{\Theta}}\left|\frac{1}{2}\ln\left|(\mathbf{Z}_2^{-1})^\top\boldsymbol{\Lambda}\left(\mathbf{Z}_2^{-1}\right)^\top\right|\right| + \sup_{\boldsymbol{\theta}\in\boldsymbol{\Theta}}\left|\frac{\nu+R}{2}\right|$$
$$\times\,\mathbb{E}\left[\sup_{\boldsymbol{\theta}\in\boldsymbol{\Theta}}\left|\ln\left(1+\frac{\left(\mathbf{Z}_1\boldsymbol{y}_t-\mathbf{X}_t\boldsymbol{\beta}-\boldsymbol{\mu}_{t|t-1}\right)^\top\mathbf{Z}_2^\top\boldsymbol{\Lambda}^{-1}\mathbf{Z}_2\left(\mathbf{Z}_1\boldsymbol{y}_t-\mathbf{X}_t\boldsymbol{\beta}-\boldsymbol{\mu}_{t|t-1}\right)}{\nu}\right)\right|\right],$$

where we note that by $|\rho_1| < 1$, $|\rho_2| < 1$ and by boundedness of $\mathbf{W}_1$ and $\mathbf{W}_2$ there exist some $Z_1^- < \det\mathbf{Z}_1 < Z_1^-$, $Z_2^- < \det\mathbf{Z}_2 < Z_2^-$, as well as it exists some $\Lambda_- < \det\boldsymbol{\Lambda} < \Lambda_+$, where $\Lambda_-, Z_1^-, Z_2^- > 0$ and $\Lambda_+, Z_1^+, Z_2^+ < \infty$. Moreover, the existence of the logarithmic moment of $(\mathbf{Z}_1\boldsymbol{y}_t-\mathbf{X}_t\boldsymbol{\beta}-\boldsymbol{\mu}_{t|t-1})^\top\mathbf{Z}_2^\top\boldsymbol{\Lambda}^{-1}\mathbf{Z}_2(\mathbf{Z}\boldsymbol{y}_t-\mathbf{X}_t\boldsymbol{\beta}-\boldsymbol{\mu}_{t|t-1})/\nu$ is ensured by Propositions 2 and 3. In particular, note that $\mathbb{E}[\sup_{\boldsymbol{\theta}\in\boldsymbol{\Theta}} |(\mathbf{Z}_1\boldsymbol{y}_t-\mathbf{X}_t\boldsymbol{\beta}-\boldsymbol{\mu}_{t|t-1})^\top\mathbf{Z}_2^\top\boldsymbol{\Lambda}^{-1}\mathbf{Z}_2(\mathbf{Z}\boldsymbol{y}_t-\boldsymbol{\mu}_{t|t-1})/\nu|^m] < \infty$ is always satisfied for some $m > 0$ and with $\nu > 0$.

Since the parameter space $\boldsymbol{\Theta}$ is compact by assumption and the likelihood function $\mathcal{L}_T(\boldsymbol{\theta})$ is continuous in $\boldsymbol{\theta}$ $\forall\boldsymbol{y}_t \in \mathbb{R}^R$ and $\mathcal{F}_t$-measurable $\forall\boldsymbol{\theta}\in\boldsymbol{\Theta}$, all the conditions of Theorem A.2.2 in White (1994) are satisfied and almost sure convergence of the second term in the right hand side of (S4) follows. The latter result also ensures the continuity in $\boldsymbol{\theta}\in\boldsymbol{\Theta}$ of the limit likelihood function $\mathcal{L}(\boldsymbol{\theta})$.

In conclusion, identifiability and uniqueness of the maximizer $\boldsymbol{\theta}_0$ of the limit in $\mathcal{L}(\boldsymbol{\theta})$ follow by the assumptions on ϕ, $\mathbf{K}$, $\boldsymbol{\Theta}$ and by Lemma C.2 in D'Innocenzo et al. (2020). Then, the strong consistency follows by White (1994, Theorem 3.4).

S5 Proof of Theorem 3.2

Standard arguments for the proof of asymptotic normality and Taylor theorem lead to the expansion of the score function about a neighborhood of $\boldsymbol{\theta}_0$,

$$\begin{aligned}\mathbf{0} = \sqrt{T}\widehat{\mathcal{L}}_T'(\hat{\boldsymbol{\theta}}_T) =& \sqrt{T}\left[\widehat{\mathcal{L}}_T'(\boldsymbol{\theta}_0) - \mathcal{L}_T'(\boldsymbol{\theta}_0)\right] + \sqrt{T}\mathcal{L}_T'(\boldsymbol{\theta}_0)\\ &+ \left[(\mathcal{L}_T''(\boldsymbol{\theta}_0) - \mathcal{L}''(\boldsymbol{\theta}_0)) + \left(\widehat{\mathcal{L}}_T''(\boldsymbol{\theta}^\star) - \mathcal{L}_T''(\boldsymbol{\theta}_0)\right) + \mathcal{L}''(\boldsymbol{\theta}_0)\right]\\ &\times\left[\sqrt{T}\left(\hat{\boldsymbol{\theta}}_T - \boldsymbol{\theta}_0\right)\right],\end{aligned} \tag{S6}$$

where $\boldsymbol{\theta}^\star$ lies on the chord between $\hat{\boldsymbol{\theta}}_T$ and $\boldsymbol{\theta}_0$, componentwise.

Considering Equation (S6), we first show that the score functions $\sqrt{T}\widehat{\mathcal{L}}'_T(\boldsymbol{\theta}_0)$ and $\sqrt{T}\mathcal{L}'_T(\boldsymbol{\theta}_0)$ have the same asymptotic distribution. We prove this argument by noting that $\mathcal{L}'_T(\boldsymbol{\theta}_0)$ obeys a central limit theorem for martingales and that the quantity $\sqrt{T}[\widehat{\mathcal{L}}'_T(\boldsymbol{\theta}_0) - \mathcal{L}'_T(\boldsymbol{\theta}_0)]$ will vanish in probability. Therefore, the desired result follows by asymptotic equivalence, see White (2001, Lemma 4.7). However, it should be noted that, as the likelihood function $\widehat{\mathcal{L}}_T(\boldsymbol{\theta})$ depends on the filter $\hat{\boldsymbol{\mu}}_{t|t-1}$, its derivative $\widehat{\mathcal{L}}'_T(\boldsymbol{\theta})$ depends both on $\hat{\boldsymbol{\mu}}_{t|t-1}$ and on the filter derivative $\partial\hat{\boldsymbol{\mu}}_{t|t-1}$. As a leading example, let us consider the derivative process with respect to the spatial parameters,

$$\frac{\partial\boldsymbol{\mu}_{t+1|t}}{\partial\rho_1} = \boldsymbol{\mathcal{X}}_t\frac{\partial\boldsymbol{\mu}_{t|t-1}}{\partial\rho_1} + \mathbf{K}\boldsymbol{r}_{1,t},$$
$$\frac{\partial\boldsymbol{\mu}_{t+1|t}}{\partial\rho_2} = \boldsymbol{\mathcal{X}}_t\frac{\partial\boldsymbol{\mu}_{t|t-1}}{\partial\rho_2} + \mathbf{K}\boldsymbol{r}_{2,t},$$

where $\boldsymbol{\mathcal{X}}_t$ is as in Proposition 3 and

$$\begin{aligned}\boldsymbol{r}_{1,t} =&\frac{\partial\boldsymbol{u}_t}{\partial\rho_1}\\ =&[2/\nu\alpha_t^{-2}(\mathbf{Z}_1\boldsymbol{y}_t - \mathbf{X}_t\boldsymbol{\beta} - \boldsymbol{\mu}_{t|t-1})(\mathbf{Z}_1\boldsymbol{y}_t - \mathbf{X}_t\boldsymbol{\beta} - \boldsymbol{\mu}_{t|t-1})^\top\mathbf{Z}_2^\top\boldsymbol{\Lambda}^{-1}\mathbf{Z}_2 - \alpha_t^{-1}]\mathbf{W}_1\boldsymbol{y}_t, \quad \text{(S7)}\\ \boldsymbol{r}_{2,t} =&\frac{\partial\boldsymbol{u}_t}{\partial\rho_2}\\ =&[2/\nu\alpha_t^{-2}(\mathbf{Z}_1\boldsymbol{y}_t - \mathbf{X}_t\boldsymbol{\beta} - \boldsymbol{\mu}_{t|t-1})^\top\mathbf{Z}_2^\top\boldsymbol{\Lambda}^{-1}\mathbf{W}_2(\mathbf{Z}_1\boldsymbol{y}_t - \mathbf{X}_t\boldsymbol{\beta} - \boldsymbol{\mu}_{t|t-1})]\\ &\times(\mathbf{Z}_1\boldsymbol{y}_t - \mathbf{X}_t\boldsymbol{\beta} - \boldsymbol{\mu}_{t|t-1}) \quad \text{(S8)}\end{aligned}$$

It is straightforward to see that both $\boldsymbol{r}_{1,t}$ and $\boldsymbol{r}_{2,t}$ in Equations (S7) and (S8) respectively, are uniformly bounded and the contraction condition imposed to the filter $\hat{\boldsymbol{\mu}}_{t|t-1}$ also implies that the nonstationary sequence $\partial\hat{\boldsymbol{\mu}}_{t|t-1}$ converges to the stationary and ergodic counterpart $\partial\boldsymbol{\mu}_{t|t-1}$. It can be shown that the same results hold for all the derivative processes. Thus,

$$\sqrt{T}\|\widehat{\mathcal{L}}'_T(\boldsymbol{\theta}_0) - \mathcal{L}'_T(\boldsymbol{\theta}_0)\| \le \sqrt{T}\left\|\begin{matrix}\frac{\partial(\widehat{\mathcal{L}}'_T(\boldsymbol{\theta}_0))}{\partial\hat{\boldsymbol{\mu}}^{\star\top}_{t|t-1}}\\ \frac{\partial(\widehat{\mathcal{L}}'_T(\boldsymbol{\theta}_0))}{\partial(\partial\hat{\boldsymbol{\mu}}^{\star\top}_{t|t-1})}\end{matrix}\right\|\left\|\begin{matrix}(\hat{\boldsymbol{\mu}}_{t|t-1}(\boldsymbol{\theta}_0) - \boldsymbol{\mu}_{t|t-1}(\boldsymbol{\theta}_0))\\ (\partial\hat{\boldsymbol{\mu}}_{t|t-1}(\boldsymbol{\theta}_0) - \partial\boldsymbol{\mu}_{t|t-1}(\boldsymbol{\theta}_0))\end{matrix}\right\|.$$

The elements in the first term on the right hand side of the above inequality are uniformly bounded, while the elements in the second term will vanish as $t \to \infty$ exponentially fast. In turn, this implies that $\sqrt{T}\|\widehat{\mathcal{L}}'_T(\boldsymbol{\theta}_0) - \mathcal{L}'_T(\boldsymbol{\theta}_0)\| \xrightarrow{\text{P}} 0$.

Let us consider the second line of Equation (S6). We note that $\widehat{\mathcal{L}}''_T(\boldsymbol{\theta})$ depends on $\hat{\boldsymbol{\mu}}_{t|t-1}$, $\partial\hat{\boldsymbol{\mu}}_{t|t-1}$ and the second derivatives $\partial^2\hat{\boldsymbol{\mu}}_{t|t-1}$. Hence, as before, we consider

$$\frac{\partial^2\boldsymbol{\mu}_{t+1|t}}{\partial\rho_1^2} = \boldsymbol{\mathcal{X}}_t\frac{\partial^2\boldsymbol{\mu}_{t|t-1}}{\partial\rho_1^2} + \mathbf{K}\left(\frac{\partial\boldsymbol{\mu}_{t|t-1}}{\partial\rho_1}\right)^\top \boldsymbol{\mathcal{C}}'_t\left(\frac{\partial\boldsymbol{\mu}_{t|t-1}}{\partial\rho_1}\right) + \mathbf{K}\boldsymbol{r}'_{1,t}, \tag{S9}$$

$$\frac{\partial^2\boldsymbol{\mu}_{t+1|t}}{\partial\rho_2^2} = \boldsymbol{\mathcal{X}}_t\frac{\partial^2\boldsymbol{\mu}_{t|t-1}}{\partial\rho_2^2} + \mathbf{K}\left(\frac{\partial\boldsymbol{\mu}_{t|t-1}}{\partial\rho_2}\right)^\top \boldsymbol{\mathcal{C}}'_t\left(\frac{\partial\boldsymbol{\mu}_{t|t-1}}{\partial\rho_2}\right) + \mathbf{K}\boldsymbol{r}'_{2,t}, \tag{S10}$$

$$\frac{\partial^2\boldsymbol{\mu}_{t+1|t}}{\partial\rho_1\partial\rho_2} = \boldsymbol{\mathcal{X}}_t\frac{\partial^2\boldsymbol{\mu}_{t|t-1}}{\partial\rho_1\partial\rho_2} + \mathbf{K}\left(\frac{\partial\boldsymbol{\mu}_{t|t-1}}{\partial\rho_1}\right)^\top \boldsymbol{\mathcal{C}}'_t\left(\frac{\partial\boldsymbol{\mu}_{t|t-1}}{\partial\rho_2}\right) + \mathbf{K}\boldsymbol{r}'_{12,t}, \tag{S11}$$

where $\boldsymbol{\mathcal{C}}'_t$ is obtained as

$$\begin{aligned}
\boldsymbol{\mathcal{C}}'_t =& \frac{\partial^2\boldsymbol{u}_t}{\partial\boldsymbol{\mu}_{t|t-1}\partial\boldsymbol{\mu}_{t|t-1}^\top} \\
=& 8/\nu^2\alpha_t^{-3}\Big\{\big[\boldsymbol{I}_R\otimes(\mathbf{Z}_1\boldsymbol{y}_t-\mathbf{X}_t\boldsymbol{\beta}-\boldsymbol{\mu}_{t|t-1})(\mathbf{Z}_1\boldsymbol{y}_t-\mathbf{X}_t\boldsymbol{\beta}-\boldsymbol{\mu}_{t|t-1})^\top\big]\mathbf{Z}_2^\top\boldsymbol{\Lambda}^{-1}\mathbf{Z}_2\Big\} \\
&\times\big[(\mathbf{Z}\boldsymbol{y}_t-\mathbf{X}_t\boldsymbol{\beta}-\boldsymbol{\mu}_{t|t-1})^\top\mathbf{Z}_2^\top\boldsymbol{\Lambda}^{-1}\mathbf{Z}_2\big] \\
&-2/\nu\alpha_t^{-2}\Big\{\big[\mathbf{Z}_2^\top\boldsymbol{\Lambda}^{-1}\mathbf{Z}_2\otimes\boldsymbol{I}_R\big] \\
&\times\big[(\mathbf{Z}_1\boldsymbol{y}_t-\mathbf{X}_t\boldsymbol{\beta}-\boldsymbol{\mu}_{t|t-1})\otimes\boldsymbol{I}_R+\boldsymbol{I}_R\otimes(\mathbf{Z}_1\boldsymbol{y}_t-\mathbf{X}_t\boldsymbol{\beta}-\boldsymbol{\mu}_{t|t-1})\big]\Big\} \\
&-2/\nu\alpha_t^{-2}\Big\{\big[\mathbf{Z}_2^\top\boldsymbol{\Lambda}^{-1}\mathbf{Z}_2\otimes\boldsymbol{I}_R\big]\big[(\mathbf{Z}\boldsymbol{y}_t-\mathbf{X}_t\boldsymbol{\beta}-\boldsymbol{\mu}_{t|t-1})\otimes\boldsymbol{I}_R\big]\Big\},
\end{aligned} \tag{S12}$$

and

$$\begin{aligned}
\boldsymbol{r}'_{1,t} =& \frac{\partial^2\boldsymbol{u}_t}{\partial\rho_1^2} \\
=& 8/\nu^2\alpha_t^{-3}\Big[(\mathbf{Z}_1\boldsymbol{y}_t-\mathbf{X}_t\boldsymbol{\beta}-\boldsymbol{\mu}_{t|t-1})\mathbf{Z}_2^\top\boldsymbol{\Lambda}^{-1}\mathbf{Z}_2\mathbf{W}_1\boldsymbol{y}_t\Big]^2(\mathbf{Z}_1\boldsymbol{y}_t-\mathbf{X}_t\boldsymbol{\beta}-\boldsymbol{\mu}_{t|t-1}) \\
&-2/\nu\alpha_t^{-2}\Big[(\mathbf{Z}_1\boldsymbol{y}_t-\mathbf{X}_t\boldsymbol{\beta}-\boldsymbol{\mu}_{t|t-1})^\top\mathbf{Z}_2^\top\boldsymbol{\Lambda}^{-1}\mathbf{Z}_2(\mathbf{W}_1\boldsymbol{y}_t)^2\Big] \\
&-2/\nu\alpha_t^{-2}\Big[\mathbf{W}_1\boldsymbol{y}_t(\mathbf{Z}_1\boldsymbol{y}_t-\mathbf{X}_t\boldsymbol{\beta}-\boldsymbol{\mu}_{t|t-1})^\top+(\mathbf{Z}_1\boldsymbol{y}_t-\mathbf{X}_t\boldsymbol{\beta}-\boldsymbol{\mu}_{t|t-1})\boldsymbol{y}_t^\top\mathbf{W}_1^\top\Big] \\
&\times\mathbf{Z}_2^\top\boldsymbol{\Lambda}^{-1}\mathbf{Z}_2\mathbf{W}_1\boldsymbol{y}_t,
\end{aligned} \tag{S13}$$

$$
\begin{aligned}
\boldsymbol{r}'_{2,t} =& \frac{\partial^2 \boldsymbol{u}_t}{\partial \rho_2^2} \\
=& \Big\{ 8/\nu^2 \alpha_t^{-3} [(\mathbf{Z}_1 \boldsymbol{y}_t - \mathbf{X}_t \boldsymbol{\beta} - \boldsymbol{\mu}_{t|t-1})^\top \mathbf{Z}_2^\top \boldsymbol{\Lambda}^{-1} \mathbf{W}_2 (\mathbf{Z}_1 \boldsymbol{y}_t - \mathbf{X}_t \boldsymbol{\beta} - \boldsymbol{\mu}_{t|t-1})]^2 \\
& - 2/\nu \alpha_t^{-2} (\mathbf{Z}_1 \boldsymbol{y}_t - \mathbf{X}_t \boldsymbol{\beta} - \boldsymbol{\mu}_{t|t-1})^\top \mathbf{W}_2^\top \boldsymbol{\Lambda}^{-1} \mathbf{W}_2 (\mathbf{Z}_1 \boldsymbol{y}_t - \mathbf{X}_t \boldsymbol{\beta} - \boldsymbol{\mu}_{t|t-1}) \Big\} \\
& \times (\mathbf{Z}_1 \boldsymbol{y}_t - \mathbf{X}_t \boldsymbol{\beta} - \boldsymbol{\mu}_{t|t-1}),
\end{aligned} \tag{S14}
$$

$$
\begin{aligned}
\boldsymbol{r}'_{12,t} =& \frac{\partial^2 \boldsymbol{u}_t}{\partial \rho_1 \partial \rho_2} \\
=& \Big\{ 8/\nu^2 \alpha_t^{-3} (\mathbf{Z}_1 \boldsymbol{y}_t - \mathbf{X}_t \boldsymbol{\beta} - \boldsymbol{\mu}_{t|t-1})^\top \mathbf{Z}_2^\top \boldsymbol{\Lambda}^{-1} \mathbf{W}_2 (\mathbf{Z}_1 \boldsymbol{y}_t - \mathbf{X}_t \boldsymbol{\beta} - \boldsymbol{\mu}_{t|t-1}) \\
& \times (\mathbf{Z}_1 \boldsymbol{y}_t - \mathbf{X}_t \boldsymbol{\beta} - \boldsymbol{\mu}_{t|t-1})^\top \mathbf{Z}_2^\top \boldsymbol{\Lambda}^{-1} \mathbf{Z}_2 \mathbf{W}_1 \boldsymbol{y}_t \\
& - 4/\nu \alpha_t^{-2} (\mathbf{Z}_1 \boldsymbol{y}_t - \mathbf{X}_t \boldsymbol{\beta} - \boldsymbol{\mu}_{t|t-1}) \mathbf{Z}_2^\top \boldsymbol{\Lambda}^{-1} \mathbf{W}_2 \mathbf{W}_1 \boldsymbol{y}_t \Big\} \\
& \times (\mathbf{Z}_1 \boldsymbol{y}_t - \mathbf{X}_t \boldsymbol{\beta} - \boldsymbol{\mu}_{t|t-1})
\end{aligned} \tag{S15}
$$

The partial derivatives in (S12), together with (S13), (S14) and (S15) are uniformly bounded. It follows from Equations (S9), (S10) and (S11) that the contraction condition on the filter also implies that $\partial^2 \hat{\boldsymbol{\mu}}_{t|t-1}$ converges to $\partial^2 \boldsymbol{\mu}_{t|t-1}$. Therefore, by employing similar arguments as in the proof of Theorem 3.1, we have $\sup_{\boldsymbol{\theta} \in \boldsymbol{\Theta}} |\widehat{\mathcal{L}}''_T(\boldsymbol{\theta}) - \mathcal{L}''_T(\boldsymbol{\theta})| \xrightarrow{\text{a.s.}} 0$ *as* $T \to \infty$. Finally that the additional condition $\mathbb{E}[||\boldsymbol{\mathcal{X}}_t \otimes \boldsymbol{\mathcal{X}}_t||] < 1$ guarantees that the likelihood second derivatives have at least one bounded moment, that is $\mathbb{E}[\mathcal{L}''_T(\boldsymbol{\theta})] < \infty$, see D'Innocenzo et al. (2020, Lemma C.11), which also entails the uniform convergence of the first summand in the second line of Equation (S6) as another application of White (1994, Theorem A.2.2). Finally, Slutsky Lemma completes the proof, see van der Vaart (1998, Lemma 2.8 (iii)). □

S6 The conditional information matrix

Let us consider the Fisher Information matrix $\mathcal{I}(\boldsymbol{\theta}_0)$ evaluated at $\boldsymbol{\theta}_0$, as in Theorem 3.2, and defined as

$$
\mathcal{I}(\boldsymbol{\theta}_0) = -\mathbb{E}\left[\left. \frac{\partial^2 \ell_t(\boldsymbol{\theta})}{\partial \boldsymbol{\theta} \partial \boldsymbol{\theta}^\top} \right|_{\boldsymbol{\theta} = \boldsymbol{\theta}_0} \right].
$$

By the law of iterated expectations, denoting $\mathbb{E}_{t-h}[Y_t] = \mathbb{E}\left[Y_t|\mathcal{F}_{t-h}\right]$,

$$\mathcal{I}(\boldsymbol{\theta}) = -\mathbb{E}\left[\mathbb{E}_{t-1}\left[\frac{\partial^2 \ell_t(\boldsymbol{\theta})}{\partial\boldsymbol{\theta}\partial\boldsymbol{\theta}^\top}\right]\right] = -\mathbb{E}\left[\mathcal{I}_t(\boldsymbol{\theta})\right]$$

where $\mathcal{I}_t(\boldsymbol{\theta})$ is the conditional information matrix defined as follows

$$\mathcal{I}_t(\boldsymbol{\theta}) = -\mathbb{E}_{t-1}\left[\frac{\partial^2 \ell_t(\boldsymbol{\theta})}{\partial\boldsymbol{\theta}\partial\boldsymbol{\theta}^\top}\right].$$

In Gaussian models, the conditional information matrix coincides with the Fisher information matrix. Otherwise (in our case, when $\nu < \infty$), $\mathcal{I}_t(\boldsymbol{\theta})$ is generally not equal to $\mathcal{I}(\boldsymbol{\theta})$. Nevertheless, in non Gaussian dynamic models, where the closed-form expression of the Fisher information matrix is typically prohibitive, inference on $\boldsymbol{\theta}_0$ can be carried out based on the analytic form of $\mathcal{I}_t(\boldsymbol{\theta})$, if available, under the same regularity conditions that lead to asymptotic normality. Increased accuracy is expected with respect to numerical approximations based on numerical derivatives that are often rather poor, see the discussion in Fiorentini et al. (2003), Bollerslev and Wooldridge (1992), based on Crowder (1976). The crucial point is that the score vector evaluated at the true parameter value $\boldsymbol{\theta}_0$ is a martingale difference sequence, so that, under correct specification and under the assumptions of Theorem 3.2, asymptotic results for martingale difference sequences apply. As

$$\mathcal{I}_t(\boldsymbol{\theta}_0) \xrightarrow{\text{a.s.}} \mathcal{I}(\boldsymbol{\theta}_0)$$

by Theorem 3.1, a consistent estimator of $\mathcal{I}(\boldsymbol{\theta}_0)$ is obtained as

$$\hat{\mathcal{I}}(\hat{\boldsymbol{\theta}}_T) = \frac{1}{T}\sum_{t=1}^{T}\hat{\mathcal{I}}_t(\hat{\boldsymbol{\theta}}_T)$$

where $\hat{\mathcal{I}}_t(\hat{\boldsymbol{\theta}}_T)$ is the conditional information matrix evaluated at the filtered sequence $\hat{\boldsymbol{\mu}}_{t|t-1}$ and at the maximum likelihood estimate $\hat{\boldsymbol{\theta}}_T$.

We now derive the conditional information matrix that we write in block form as

$$\mathcal{I}_t(\boldsymbol{\theta}) = \begin{bmatrix}
\mathcal{I}_t^{(\nu)}(\boldsymbol{\theta}) & * & * & * & * & * & * \\
\mathcal{I}_t^{(\mathrm{v}(\boldsymbol{\Lambda}),\nu)}(\boldsymbol{\theta}) & \mathcal{I}_t^{(\mathrm{v}(\boldsymbol{\Lambda}))}(\boldsymbol{\theta}) & * & * & * & * & * \\
\mathcal{I}_t^{(\rho_1,\nu)}(\boldsymbol{\theta}) & \mathcal{I}_t^{(\rho_1,\mathrm{v}(\boldsymbol{\Lambda}))}(\boldsymbol{\theta}) & \mathcal{I}_t^{(\rho_1)}(\boldsymbol{\theta}) & * & * & * & * \\
\mathcal{I}_t^{(\rho_2,\nu)}(\boldsymbol{\theta}) & \mathcal{I}_t^{(\rho_2,\mathrm{v}(\boldsymbol{\Lambda}))}(\boldsymbol{\theta}) & \mathcal{I}_t^{(\rho_2,\rho_1)}(\boldsymbol{\theta}) & \mathcal{I}_t^{(\rho_2)}(\boldsymbol{\theta}) & * & * & * \\
\mathbf{0} & \mathbf{0} & \mathcal{I}_t^{(\boldsymbol{\beta},\rho_1)}(\boldsymbol{\theta}) & \mathbf{0} & \mathcal{I}_t^{(\boldsymbol{\beta})}(\boldsymbol{\theta}) & * & * \\
\mathcal{I}_t^{(\phi,\nu)}(\boldsymbol{\theta}) & \mathcal{I}_t^{(\phi,\mathrm{v}(\boldsymbol{\Lambda}))}(\boldsymbol{\theta}) & \mathcal{I}_t^{(\phi,\rho_1)}(\boldsymbol{\theta}) & \mathcal{I}_t^{(\phi,\rho_2)}(\boldsymbol{\theta}) & \mathbf{0} & \mathcal{I}_t^{(\phi)}(\boldsymbol{\theta}) & * \\
\mathcal{I}_t^{(\mathrm{v}(\mathbf{K}),\nu)}(\boldsymbol{\theta}) & \mathcal{I}_t^{(\mathrm{v}(\mathbf{K}),\mathrm{v}(\boldsymbol{\Lambda}))}(\boldsymbol{\theta}) & \mathcal{I}_t^{(\mathrm{v}(\mathbf{K}),\rho_1)}(\boldsymbol{\theta}) & \mathcal{I}_t^{(\mathrm{v}(\mathbf{K}),\rho_2)}(\boldsymbol{\theta}) & \mathbf{0} & \mathcal{I}_t^{(\mathrm{v}(\mathbf{K}),\phi)}(\boldsymbol{\theta}) & \mathcal{I}_t^{(\mathrm{v}(\mathbf{K}))}(\boldsymbol{\theta})
\end{bmatrix}$$

where the symbols $*$ in correspondence of the (i,j)-th element denote that the transposed of the corresponding (j,i)-th element is considered. As in the proof of Theorem 3.2, we derive, as a leading example, the information with respect to the spatial parameters, that is $\mathcal{I}_t^{(\rho_1,\rho_2)}(\boldsymbol{\theta})$. The other components or blocks can be obtained analogously.

Some notation follows. Let $\mathrm{v}(\mathbf{L}) := \mathrm{vec}(\mathbf{L})$ denote the vector operator applied to the matrix $\mathbf{L}$ to stack in a vector the columns of $\mathbf{L}$, $\mathrm{vech}(\mathbf{L})$ denote the vector operator applied to the lower-triangular part of $\mathbf{L}$, diagonal elements included, and $\mathrm{de\,vec}(\mathrm{vec}(\mathbf{L})) = \mathbf{L}$. Let $\boldsymbol{\mathcal{D}}_R \in \mathbb{R}^{R^2\times\frac{R(R+1)}{2}}$ denote the duplication matrix, which satisfies $\boldsymbol{D}_R\,\mathrm{vech}(\mathbf{L}) = \mathrm{vec}(\mathbf{L})$, and $\boldsymbol{\mathcal{K}}_{RR} \in \mathbb{R}^{R^2\times R^2}$ denote the commutation matrix, which satisfies $\boldsymbol{\mathcal{K}}_{RR}\,\mathrm{vec}(\mathbf{L}) = \mathrm{vec}(\mathbf{L}^\top)$. Finally, $\psi'(z) = \partial^2\log\Gamma(z)/\partial z^2$ is the trigamma function of z, the symbol $\otimes$ denotes the Kronecker product and $\mathrm{tr}(A)$ indicates the trace of A.

The second derivative (or the Hessian) of the log-likelihood with respect to the parameters (ρ_1,ρ_2), can be written as follows:

$$\mathcal{H}_t^{(\rho_1,\rho_2)}(\boldsymbol{\theta}) = \frac{\partial^2\ell_t(\boldsymbol{\theta})}{\partial\rho_1\partial\rho_2} + \left(\frac{\partial\boldsymbol{\mu}_{t|t-1}}{\partial\rho_1}\right)^\top \frac{\partial^2\ell_t(\boldsymbol{\theta})}{\partial\boldsymbol{\mu}_{t|t-1}\partial\boldsymbol{\mu}_{t|t-1}^\top}\left(\frac{\partial\boldsymbol{\mu}_{t|t-1}}{\partial\rho_2}\right) + \frac{\partial\ell_t(\boldsymbol{\theta})}{\partial\boldsymbol{\mu}_{t|t-1}^\top}\frac{\partial^2\boldsymbol{\mu}_{t|t-1}}{\partial\rho_1\partial\rho_2}, \tag{S16}$$

where

$$\begin{aligned}\frac{\partial^2\ell_t(\boldsymbol{\theta})}{\partial\rho_1\partial\rho_2} =& 2\frac{\nu+R}{\nu^2}\alpha_t^{-2}(\mathbf{Z}_1\boldsymbol{y}_t - \mathbf{X}_t\boldsymbol{\beta} - \boldsymbol{\mu}_{t|t-1})^\top\mathbf{Z}_2\boldsymbol{\Lambda}^{-1}\mathbf{W}_2(\mathbf{Z}_1\boldsymbol{y}_t - \mathbf{X}_t\boldsymbol{\beta} - \boldsymbol{\mu}_{t|t-1})\\ &\times \boldsymbol{y}_t^\top\mathbf{W}_1^\top\mathbf{Z}_2\boldsymbol{\Lambda}^{-1}\mathbf{Z}_2(\mathbf{Z}_1\boldsymbol{y}_t - \mathbf{X}_t\boldsymbol{\beta} - \boldsymbol{\mu}_{t|t-1})\\ &- \frac{\nu+R}{\nu}\alpha_t^{-1}\boldsymbol{y}_t^\top\mathbf{W}_1^\top\mathbf{W}_2^\top\boldsymbol{\Lambda}^{-1}\mathbf{Z}_2(\mathbf{Z}_1\boldsymbol{y}_t - \mathbf{X}_t\boldsymbol{\beta} - \boldsymbol{\mu}_{t|t-1})\\ &- \frac{\nu+R}{\nu}\alpha_t^{-1}\boldsymbol{y}_t^\top\mathbf{W}_1^\top\mathbf{Z}_2^\top\boldsymbol{\Lambda}^{-1}\mathbf{W}_2(\mathbf{Z}_1\boldsymbol{y}_t - \mathbf{X}_t\boldsymbol{\beta} - \boldsymbol{\mu}_{t|t-1}),\end{aligned} \tag{S17}$$

$$\begin{aligned}\frac{\partial^2\ell_t(\boldsymbol{\theta})}{\partial\boldsymbol{\mu}_{t|t-1}\partial\boldsymbol{\mu}_{t|t-1}^\top} =& 2\frac{\nu+N}{\nu^2}\alpha_t^{-2}\boldsymbol{\Lambda}^{-1}(\mathbf{Z}_1\boldsymbol{y}_t - \mathbf{X}_t\boldsymbol{\beta} - \boldsymbol{\mu}_{t|t-1})(\mathbf{Z}_1\boldsymbol{y}_t - \mathbf{X}_t\boldsymbol{\beta} - \boldsymbol{\mu}_{t|t-1})^\top\boldsymbol{\Lambda}^{-1}\\ &- \frac{\nu+N}{\nu}\alpha_t^{-1}\boldsymbol{\Lambda}^{-1}\end{aligned}$$

and

$$\frac{\partial\ell_t(\boldsymbol{\theta})}{\partial\boldsymbol{\mu}_{t|t-1}^\top} = \frac{\nu+R}{\nu}\boldsymbol{u}_t^\top\boldsymbol{\Lambda}^{-1}\mathbf{Z}_2,$$

while $\partial\boldsymbol{\mu}_{t|t-1}/\partial\rho_1$, $\partial\boldsymbol{\mu}_{t|t-1}/\partial\rho_2$ and $\partial^2\boldsymbol{\mu}_{t|t-1}/\partial\rho_1\partial\rho_2$ are defined as in (S9), (S10) and (S11), respectively.

Let us write $\mathbf{Z}_1\boldsymbol{y}_t - \mathbf{X}_t\boldsymbol{\beta} - \boldsymbol{\mu}_{t|t-1} = \mathbf{Z}_2^{-1}\boldsymbol{\eta}_t$ and consider the following stochastic representation of the elliptically distributed Student-t variable (see Fang et al. 1990)

$$\boldsymbol{\eta}_t = \sqrt{\frac{r_t}{s_t/\nu}}\mathbf{Z}_1^{-1}\mathbf{Z}_2^{-1}\boldsymbol{\Lambda}^{1/2}\mathbf{q}_t,$$

where r_t, s_t and $\mathbf{q}_t$ are mutually independent random variables, such that $r_t \sim \chi^2(R)$, $s_t \sim \chi^2(\nu)$, with $\chi^2(r)$ indicating a chi-square distributed random variable with r degrees of freedom, and $\mathbf{q}_t$ is uniformly distributed on the unit sphere in $\mathbb{R}^R$. Like in Fiorentini et al. (2003), Equation (S17) may be written in terms of $\mathbf{q}_t$ as follows

$$\begin{aligned}\frac{\partial^2\ell_t(\boldsymbol{\theta})}{\partial\rho_1\partial\rho_2} =& 2(\nu+R)(1-\alpha_t^{-2})\operatorname{vec}(\boldsymbol{\Lambda}^{-1/2}\mathbf{W}_2\mathbf{Z}_2^{-1}\boldsymbol{\Lambda}^{1/2})(\mathbf{q}_t\mathbf{q}_t^\top \otimes \mathbf{q}_t\mathbf{q}_t^\top)\\ &\times \operatorname{vec}(\boldsymbol{\Lambda}^{1/2}(\mathbf{Z}_2^{-1})^\top(\mathbf{Z}_1^{-1})^\top\mathbf{W}_1^\top\mathbf{Z}_2^\top\boldsymbol{\Lambda}^{-1/2})\\ &-(\nu+R)(1-\alpha_t^{-1})(\mathbf{q}_t\otimes\mathbf{q}_t)^\top\operatorname{vec}(\boldsymbol{\Lambda}^{1/2}(\mathbf{Z}_2^{-1})^\top(\mathbf{Z}_1^{-1})^\top\mathbf{W}_1^\top\mathbf{Z}_2^\top\boldsymbol{\Lambda}^{-1}\mathbf{W}_2\mathbf{Z}_2^{-1}\boldsymbol{\Lambda}^{1/2})\\ &-(\nu+R)(1-\alpha_t^{-1})(\mathbf{q}_t\otimes\mathbf{q}_t)^\top\operatorname{vec}(\boldsymbol{\Lambda}^{1/2}(\mathbf{Z}_2^{-1})^\top(\mathbf{Z}_1^{-1})^\top\mathbf{W}_1^\top\mathbf{W}_2^\top\boldsymbol{\Lambda}^{-1/2}).\end{aligned}$$

Based on the above representation, to compute the negative conditional expectation of the Hessian in (S16), one only needs to compute the second and fourth unconditional moments of $\mathbf{q}_t$, that can be found in the Proof of Proposition 1 of Fiorentini et al. (2003), and the first and second moments of $b_t = 1-\alpha_t^{-1}$, that is a Beta $(R/2, \nu/2)$ distributed random variable.

Thus, we obtain

$$\begin{aligned}-\mathbb{E}_{t-1}\left[\frac{\partial^2\ell_t(\boldsymbol{\theta})}{\partial\rho_1\partial\rho_2}\right] =& \frac{-2}{\nu+R+2}\operatorname{vec}(\boldsymbol{\Lambda}^{-1/2}\mathbf{W}_2\mathbf{Z}_2^{-1}\boldsymbol{\Lambda}^{1/2})[\boldsymbol{I}_{R^2}+\boldsymbol{\mathcal{K}}_{RR}+(\operatorname{vec}\boldsymbol{I}_R)(\operatorname{vec}\boldsymbol{I}_R)^\top]\\ &\times\operatorname{vec}(\boldsymbol{\Lambda}^{1/2}(\mathbf{Z}_2^{-1})^\top(\mathbf{Z}_1^{-1})^\top\mathbf{W}_1^\top\mathbf{Z}_2^\top\boldsymbol{\Lambda}^{-1/2})\\ &+\operatorname{tr}(\boldsymbol{\Lambda}(\mathbf{Z}_2^{-1})^\top(\mathbf{Z}_1^{-1})^\top\mathbf{W}_1^\top\mathbf{Z}_2^\top\boldsymbol{\Lambda}^{-1}\mathbf{W}_2\mathbf{Z}_2^{-1})\\ &+\operatorname{tr}((\mathbf{Z}_2^{-1})^\top(\mathbf{Z}_1^{-1})^\top\mathbf{W}_1^\top\mathbf{W}_2^\top).\end{aligned}$$

Similar arguments lead to

$$-\mathbb{E}_{t-1}\left[\frac{\partial^2\ell_t(\boldsymbol{\theta})}{\partial\boldsymbol{\mu}_{t|t-1}\partial\boldsymbol{\mu}_{t|t-1}^\top}\right] = \frac{\nu+R}{\nu+R+2}\boldsymbol{\Lambda}^{-1},$$

and to

$$\frac{\partial \ell_t(\boldsymbol{\theta})}{\partial \boldsymbol{\mu}_{t|t-1}^\top} = \mathbf{0}_R^\top.$$

The latter result is crucial, in that it implies that all the second derivatives of the Hessian in (S16) will cancel out, since $\partial \boldsymbol{\mu}_{t|t-1}/\partial \rho_1$, $\partial \boldsymbol{\mu}_{t|t-1}/\partial \rho_2$ and $\partial^2 \boldsymbol{\mu}_{t|t-1}/\partial \rho_1 \partial \rho_2$ are $\mathcal{F}_{t-1}$-measurable. In conclusion, we have just proved that

$$\begin{aligned}
\mathcal{I}_t^{(\rho_1,\rho_2)}(\boldsymbol{\theta}) =& - \mathbb{E}_{t-1}[\mathcal{H}_t^{(\rho_1,\rho_2)}(\boldsymbol{\theta})] \\
=& \operatorname{tr}(\boldsymbol{\Lambda}(\mathbf{Z}_2^{-1})^\top (\mathbf{Z}_1^{-1})^\top \mathbf{W}_1^\top \mathbf{Z}_2^\top \boldsymbol{\Lambda}^{-1} \mathbf{W}_2 \mathbf{Z}_2^{-1}) + \operatorname{tr}((\mathbf{Z}_2^{-1})^\top (\mathbf{Z}_1^{-1})^\top \mathbf{W}_1^\top \mathbf{W}_2 \mathbf{Z}_2^{-1}) \\
& - \frac{2}{\nu+R+2} \operatorname{vec}(\boldsymbol{\Lambda}^{-1/2} \mathbf{W}_2 \mathbf{Z}_2^{-1} \boldsymbol{\Lambda}^{1/2})^\top [\boldsymbol{I}_{R^2} + \boldsymbol{\mathcal{K}}_{RR} + (\operatorname{vec} \boldsymbol{I}_R)(\operatorname{vec} \boldsymbol{I}_R)^\top] \times \\
& \times \operatorname{vec}(\boldsymbol{\Lambda}^{1/2} (\mathbf{Z}_2^{-1})^\top (\mathbf{Z}_1^{-1})^\top \mathbf{W}_1^\top \mathbf{Z}_2^\top \boldsymbol{\Lambda}^{-1/2}) \frac{\nu+R}{\nu+R+2} \left(\frac{\partial \boldsymbol{\mu}_{t|t-1}}{\partial \rho_1} \right)^\top \mathbf{Z}_2^\top \boldsymbol{\Lambda}^{-1} \mathbf{Z}_2 \left(\frac{\partial \boldsymbol{\mu}_{t|t-1}}{\partial \rho_2} \right).
\end{aligned}$$

All the other blocks or terms in $\mathcal{I}_t(\boldsymbol{\theta})$ are derived with a similar procedure and are reported here in the following:

$$\begin{aligned}
\mathcal{I}_t^{(\nu)}(\boldsymbol{\theta}) =& \frac{1}{4} \left[\psi' \left(\frac{\nu}{2} \right) - \psi' \left(\frac{\nu+R}{2} \right) - \frac{2R(\nu+R+4)}{\nu(\nu+R)(\nu+R+2)} \right] \\
& + \frac{\nu+R}{\nu+R+2} \left(\frac{\partial \boldsymbol{\mu}_{t|t-1}}{\partial \nu} \right)^\top \mathbf{Z}_2^\top \boldsymbol{\Lambda}^{-1} \mathbf{Z}_2 \left(\frac{\partial \boldsymbol{\mu}_{t|t-1}}{\partial \nu} \right),
\end{aligned}$$

$$\begin{aligned}
\mathcal{I}_t^{(\mathrm{v}(\boldsymbol{\Lambda}),\nu)}(\boldsymbol{\theta}) =& - \frac{1}{(\nu+R)(\nu+R+2)} \boldsymbol{\mathcal{D}}_R^\top (\operatorname{vech}(\boldsymbol{\Lambda}^{-1})) \\
& + \frac{\nu+R}{\nu+R+2} \left(\frac{\partial \boldsymbol{\mu}_{t|t-1}}{\partial (\operatorname{vec}(\boldsymbol{\Lambda}))^\top} \right)^\top \mathbf{Z}_2^\top \boldsymbol{\Lambda}^{-1} \mathbf{Z}_2 \left(\frac{\partial \boldsymbol{\mu}_{t|t-1}}{\partial \nu} \right),
\end{aligned}$$

$$\begin{aligned}
\mathcal{I}_t^{(\rho_1,\nu)}(\boldsymbol{\theta}) =& - \frac{2}{(\nu+R)(\nu+R+2)} \operatorname{tr}(\mathbf{W}_1 \mathbf{Z}_1^{-1}) \\
& + \frac{\nu+R}{\nu+R+2} \left(\frac{\partial \boldsymbol{\mu}_{t|t-1}}{\partial \rho_1} \right)^\top \mathbf{Z}_2^\top \boldsymbol{\Lambda}^{-1} \mathbf{Z}_2 \left(\frac{\partial \boldsymbol{\mu}_{t|t-1}}{\partial \nu} \right),
\end{aligned}$$

$$
\begin{aligned}
\mathcal{I}_t^{(\rho_2,\nu)}(\boldsymbol{\theta}) =& -\frac{2}{(\nu+R)(\nu+R+2)}\operatorname{tr}(\mathbf{W}_2\mathbf{Z}_2^{-1}) \\
&+\frac{\nu+R}{\nu+R+2}\left(\frac{\partial\boldsymbol{\mu}_{t|t-1}}{\partial\rho_2}\right)^\top\mathbf{Z}_2^\top\boldsymbol{\Lambda}^{-1}\mathbf{Z}_2\left(\frac{\partial\boldsymbol{\mu}_{t|t-1}}{\partial\nu}\right),
\end{aligned}
$$

$$
\begin{aligned}
\mathcal{I}_t^{(\mathrm{v}(\boldsymbol{\Lambda}))}(\boldsymbol{\theta}) =& \frac{\nu+R}{2(\nu+R+2)}\boldsymbol{\mathcal{D}}_R^\top(\boldsymbol{\Lambda}^{-1}\otimes\boldsymbol{\Lambda}^{-1})\boldsymbol{\mathcal{D}}_R \\
&-\frac{1}{2(\nu+R+2)}\boldsymbol{\mathcal{D}}_R^\top(\operatorname{vech}(\boldsymbol{\Lambda}^{-1}))(\operatorname{vech}(\boldsymbol{\Lambda}^{-1}))^\top\boldsymbol{\mathcal{D}}_R \\
&+\frac{\nu+R}{\nu+R+2}\left(\frac{\partial\boldsymbol{\mu}_{t|t-1}}{\partial(\operatorname{vech}(\boldsymbol{\Lambda}))^\top}\right)^\top\mathbf{Z}_2^\top\boldsymbol{\Lambda}^{-1}\mathbf{Z}_2\left(\frac{\partial\boldsymbol{\mu}_{t|t-1}}{\partial(\operatorname{vech}(\boldsymbol{\Lambda}))^\top}\right),
\end{aligned}
$$

$$
\begin{aligned}
\mathcal{I}_t^{(\rho_1,\mathrm{v}(\boldsymbol{\Lambda}))}(\boldsymbol{\theta}) =& (\operatorname{vec}\boldsymbol{I}_R)^\top(\boldsymbol{\Lambda}^{-1/2}\otimes\mathbf{W}_1^\top\mathbf{Z}_2^\top\boldsymbol{\Lambda}^{-1}) \\
&-\frac{1}{\nu+R+2}\operatorname{vec}(\boldsymbol{\Lambda}^{1/2}(\mathbf{Z}_1^{-1})^\top(\mathbf{Z}_2^{-1})^\top\mathbf{W}_1^\top\mathbf{Z}_2^\top\boldsymbol{\Lambda}^{-1/2})^\top \\
&\qquad\times[\boldsymbol{I}_{R^2}+\boldsymbol{\mathcal{K}}_{RR}+(\operatorname{vec}\boldsymbol{I}_R)(\operatorname{vec}\boldsymbol{I}_R)^\top](\boldsymbol{\Lambda}^{-1/2}\otimes\boldsymbol{\Lambda}^{-1/2}) \\
&+\frac{\nu+R}{\nu+R+2}\left(\frac{\partial\boldsymbol{\mu}_{t|t-1}}{\partial\rho_1}\right)^\top\mathbf{Z}_2^\top\boldsymbol{\Lambda}^{-1}\mathbf{Z}_2\left(\frac{\partial\boldsymbol{\mu}_{t|t-1}}{\partial(\operatorname{vech}(\boldsymbol{\Lambda}))^\top}\right),
\end{aligned}
$$

$$
\begin{aligned}
\mathcal{I}_t^{(\rho_2,\mathrm{v}(\boldsymbol{\Lambda}))}(\boldsymbol{\theta}) =& (\operatorname{vec}\boldsymbol{I}_R)^\top(\boldsymbol{\Lambda}^{1/2}(\mathbf{Z}_2^{-1})^\top\mathbf{W}_2^\top\boldsymbol{\Lambda}^{-1}\otimes\boldsymbol{\Lambda}^{-1/2}) \\
&-\frac{1}{\nu+R+2}\operatorname{vec}(\boldsymbol{\Lambda}^{-1/2}\mathbf{W}_2\mathbf{Z}_2^{-1}\boldsymbol{\Lambda}^{1/2})^\top \\
&\qquad\times[\boldsymbol{I}_{R^2}+\boldsymbol{\mathcal{K}}_{RR}+(\operatorname{vec}\boldsymbol{I}_R)(\operatorname{vec}\boldsymbol{I}_R)^\top](\boldsymbol{\Lambda}^{-1/2}\otimes\boldsymbol{\Lambda}^{-1/2}) \\
&+\frac{\nu+R}{\nu+R+2}\left(\frac{\partial\boldsymbol{\mu}_{t|t-1}}{\partial\rho_2}\right)^\top\mathbf{Z}_2^\top\boldsymbol{\Lambda}^{-1}\mathbf{Z}_2\left(\frac{\partial\boldsymbol{\mu}_{t|t-1}}{\partial(\operatorname{vech}(\boldsymbol{\Lambda}))^\top}\right),
\end{aligned}
$$

$$
\begin{aligned}
\mathcal{I}_t^{(\rho_1)}(\boldsymbol{\theta}) =& \operatorname{tr}(\mathbf{W}_1\mathbf{Z}_1^{-1})^2+\operatorname{tr}(\boldsymbol{\Lambda}(\mathbf{Z}_2^{-1})^\top(\mathbf{Z}_1^{-1})^\top\mathbf{W}_1^\top\mathbf{Z}_2^\top\boldsymbol{\Lambda}^{-1}\mathbf{Z}_2^{-1}\mathbf{W}_1\mathbf{Z}_1^{-1}\mathbf{Z}_2^{-1}) \\
&+\Big\{(\boldsymbol{\beta}\otimes\boldsymbol{\beta})^\top(\mathbf{X}_t\otimes\mathbf{X}_t)^\top]+(\boldsymbol{\mu}_{t|t-1}\otimes\boldsymbol{\mu}_{t|t-1})^\top
\end{aligned}
$$

$$+ (\boldsymbol{\mu}_{t|t-1} \otimes \mathbf{X}_t\boldsymbol{\beta})^\top + (\mathbf{X}_t\boldsymbol{\beta} \otimes \boldsymbol{\mu}_{t|t-1})^\top \Big\} \operatorname{vec}((\mathbf{Z}_1^{-1})^\top \mathbf{W}_1^\top \mathbf{Z}_2^\top \boldsymbol{\Lambda}^{-1}\mathbf{Z}_2\mathbf{W}_1\mathbf{Z}_1^{-1})$$

$$- \frac{2}{\nu + R + 2} \operatorname{vec}(\boldsymbol{\Lambda}^{-1/2}\mathbf{Z}_2\mathbf{W}_1\mathbf{Z}_1^{-1})$$

$$\times \Big\{ (\boldsymbol{I}_R \otimes \boldsymbol{\beta}^\top \mathbf{X}_t \mathbf{X}_t^\top \boldsymbol{\beta})^\top + (\boldsymbol{I}_R \otimes \mathbf{X}_t\boldsymbol{\beta}\boldsymbol{\mu}_{t|t-1}^\top)^\top + (\boldsymbol{I}_R \otimes \boldsymbol{\mu}_{t|t-1}\boldsymbol{\beta}^\top \mathbf{X}_t^\top])^\top$$

$$+ (\boldsymbol{I}_R \otimes \boldsymbol{\mu}_{t|t-1}\boldsymbol{\mu}_{t|t-1}^\top)^\top + [\boldsymbol{I}_{R^2} + \boldsymbol{\mathcal{K}}_{RR} + (\operatorname{vec}\boldsymbol{I}_R)(\operatorname{vec}\boldsymbol{I}_R)^\top] \Big\}$$

$$\times \operatorname{vec}((\mathbf{Z}_1^{-1})^\top \mathbf{W}_1^\top \mathbf{Z}_2^\top \boldsymbol{\Lambda}^{-1/2})$$

$$+ \frac{\nu + R}{\nu + R + 2} \left(\frac{\partial \boldsymbol{\mu}_{t|t-1}}{\partial \rho_1} \right)^\top \mathbf{Z}_2^\top \boldsymbol{\Lambda}^{-1} \mathbf{Z}_2 \left(\frac{\partial \boldsymbol{\mu}_{t|t-1}}{\partial \rho_1} \right),$$

$$\begin{aligned}
\mathcal{I}_t^{(\rho_2)}(\boldsymbol{\theta}) = & \operatorname{tr}(\mathbf{W}_2\mathbf{Z}_2^{-1})^2 + \operatorname{tr}(\boldsymbol{\Lambda}(\mathbf{Z}_2^{-1})^\top \mathbf{W}_2^\top \boldsymbol{\Lambda}^{-1}\mathbf{W}_2\mathbf{Z}_2^{-1}) \\
& - \frac{2}{\nu + R + 2} \operatorname{vec}(\boldsymbol{\Lambda}^{-1/2}\mathbf{W}_2\mathbf{Z}_2^{-1}\boldsymbol{\Lambda}^{1/2})^\top \\
& \times [\boldsymbol{I}_{R^2} + \boldsymbol{\mathcal{K}}_{RR} + (\operatorname{vec}\boldsymbol{I}_R)(\operatorname{vec}\boldsymbol{I}_R)^\top] \\
& \times \operatorname{vec}(\boldsymbol{\Lambda}^{-1/2}\mathbf{W}_2\mathbf{Z}_2^{-1}\boldsymbol{\Lambda}^{1/2}) \\
& + \frac{\nu + R}{\nu + R + 2} \left(\frac{\partial \boldsymbol{\mu}_{t|t-1}}{\partial \rho_2} \right)^\top \mathbf{Z}_2^\top \boldsymbol{\Lambda}^{-1} \mathbf{Z}_2 \left(\frac{\partial \boldsymbol{\mu}_{t|t-1}}{\partial \rho_2} \right),
\end{aligned}$$

$$\begin{aligned}
\mathcal{I}_t^{(\boldsymbol{\beta})}(\boldsymbol{\theta}) = & \frac{\nu + R}{\nu + R + 2} \operatorname{de vec} \left((\mathbf{X}_t \otimes \mathbf{X}_t)^\top \operatorname{vec}(\mathbf{Z}_2^\top \boldsymbol{\Lambda}^{-1}\mathbf{Z}_2) \right) \\
& + \frac{\nu + R}{\nu + R + 2} \left(\frac{\partial \boldsymbol{\mu}_{t|t-1}}{\partial \boldsymbol{\beta}^\top} \right)^\top \mathbf{Z}_2^\top \boldsymbol{\Lambda}^{-1} \mathbf{Z}_2 \left(\frac{\partial \boldsymbol{\mu}_{t|t-1}}{\partial \boldsymbol{\beta}^\top} \right),
\end{aligned}$$

$$\begin{aligned}
\mathcal{I}_t^{(\boldsymbol{\beta},\rho_1)}(\boldsymbol{\theta}) = & \frac{\nu + R}{\nu + R + 2} (\boldsymbol{\beta}^\top \otimes \boldsymbol{I}_R)(\mathbf{X}_t \otimes \mathbf{X}_t)^\top \operatorname{vec}(\mathbf{Z}_2^\top \boldsymbol{\Lambda}^{-1}\mathbf{Z}_2\mathbf{W}_1\mathbf{Z}_1^{-1}) \\
& + \frac{\nu + R}{\nu + R + 2} \mathbf{X}_t^\top \mathbf{Z}_2^\top \boldsymbol{\Lambda}^{-1}\mathbf{Z}_2\mathbf{W}_1\mathbf{Z}_1^{-1}\boldsymbol{\mu}_{t|t-1} \\
& + \frac{\nu + R}{\nu + R + 2} \left(\frac{\partial \boldsymbol{\mu}_{t|t-1}}{\partial \boldsymbol{\beta}^\top} \right)^\top \mathbf{Z}_2^\top \boldsymbol{\Lambda}^{-1} \mathbf{Z}_2 \left(\frac{\partial \boldsymbol{\mu}_{t|t-1}}{\partial \rho_1} \right),
\end{aligned}$$

$$\mathcal{I}_t^{(\phi,\nu)}(\boldsymbol{\theta}) = \frac{\nu+R}{\nu+R+2}\left(\frac{\partial \boldsymbol{\mu}_{t|t-1}}{\partial \phi}\right)^{\top} \mathbf{Z}_2^{\top} \boldsymbol{\Lambda}^{-1} \mathbf{Z}_2 \left(\frac{\partial \boldsymbol{\mu}_{t|t-1}}{\partial \nu}\right),$$

$$\mathcal{I}_t^{(\phi,\mathrm{v}(\boldsymbol{\Lambda}))}(\boldsymbol{\theta}) = \frac{\nu+R}{\nu+R+2}\left(\frac{\partial \boldsymbol{\mu}_{t|t-1}}{\partial \phi}\right)^{\top} \mathbf{Z}_2^{\top} \boldsymbol{\Lambda}^{-1} \mathbf{Z}_2 \left(\frac{\partial \boldsymbol{\mu}_{t|t-1}}{\partial (\mathrm{vech}(\boldsymbol{\Lambda}))^{\top}}\right),$$

$$\mathcal{I}_t^{(\phi,\rho_1)}(\boldsymbol{\theta}) = \frac{\nu+R}{\nu+R+2}\left(\frac{\partial \boldsymbol{\mu}_{t|t-1}}{\partial \phi}\right)^{\top} \mathbf{Z}_2^{\top} \boldsymbol{\Lambda}^{-1} \mathbf{Z}_2 \left(\frac{\partial \boldsymbol{\mu}_{t|t-1}}{\partial \rho_1}\right),$$

$$\mathcal{I}_t^{(\phi,\rho_2)}(\boldsymbol{\theta}) = \frac{\nu+R}{\nu+R+2}\left(\frac{\partial \boldsymbol{\mu}_{t|t-1}}{\partial \phi}\right)^{\top} \mathbf{Z}_2^{\top} \boldsymbol{\Lambda}^{-1} \mathbf{Z}_2 \left(\frac{\partial \boldsymbol{\mu}_{t|t-1}}{\partial \rho_2}\right),$$

$$\mathcal{I}_t^{(\mathrm{v}(\mathbf{K}),\mathrm{v}(\boldsymbol{\Lambda}))}(\boldsymbol{\theta}) = \frac{\nu+N}{\nu+N+2}\left(\frac{\partial \boldsymbol{\mu}_{t|t-1}}{\partial (\mathrm{vec}\,\mathbf{K})^{\top}}\right)^{\top} \mathbf{Z}_2^{\top} \boldsymbol{\Lambda}^{-1} \mathbf{Z}_2 \left(\frac{\partial \boldsymbol{\mu}_{t|t-1}}{\partial (\mathrm{vech}(\boldsymbol{\Lambda}))^{\top}}\right),$$

$$\mathcal{I}_t^{(\mathrm{v}(\mathbf{K}),\nu)}(\boldsymbol{\theta}) = \frac{\nu+R}{\nu+R+2}\left(\frac{\partial \boldsymbol{\mu}_{t|t-1}}{\partial (\mathrm{vec}\,\mathbf{K})^{\top}}\right)^{\top} \mathbf{Z}_2^{\top} \boldsymbol{\Lambda}^{-1} \mathbf{Z}_2 \left(\frac{\partial \boldsymbol{\mu}_{t|t-1}}{\partial \nu}\right),$$

$$\mathcal{I}_t^{(\mathrm{v}(\mathbf{K}),\rho_1)}(\boldsymbol{\theta}) = \frac{\nu+R}{\nu+R+2}\left(\frac{\partial \boldsymbol{\mu}_{t|t-1}}{\partial (\mathrm{vec}\,\mathbf{K})^{\top}}\right)^{\top} \mathbf{Z}_2^{\top} \boldsymbol{\Lambda}^{-1} \mathbf{Z}_2 \left(\frac{\partial \boldsymbol{\mu}_{t|t-1}}{\partial \rho_1}\right),$$

$$\mathcal{I}_t^{(\mathrm{v}(\mathbf{K}),\rho_2)}(\boldsymbol{\theta}) = \frac{\nu+R}{\nu+R+2}\left(\frac{\partial \boldsymbol{\mu}_{t|t-1}}{\partial (\mathrm{vec}\,\mathbf{K})^{\top}}\right)^{\top} \mathbf{Z}_2^{\top} \boldsymbol{\Lambda}^{-1} \mathbf{Z}_2 \left(\frac{\partial \boldsymbol{\mu}_{t|t-1}}{\partial \rho_2}\right),$$

$$\mathcal{I}_t^{\phi}(\boldsymbol{\theta}) = \frac{\nu+R}{\nu+R+2}\left(\frac{\partial \boldsymbol{\mu}_{t|t-1}}{\partial \phi}\right)^{\top} \mathbf{Z}_2^{\top} \boldsymbol{\Lambda}^{-1} \mathbf{Z}_2 \left(\frac{\partial \boldsymbol{\mu}_{t|t-1}}{\partial \phi}\right),$$

$$\mathcal{I}_t^{(\phi,\mathrm{v}(\mathbf{K}))}(\boldsymbol{\theta}) = \frac{\nu+R}{\nu+R+2}\left(\frac{\partial \boldsymbol{\mu}_{t|t-1}}{\partial \phi}\right)^{\top} \mathbf{Z}_2^{\top} \boldsymbol{\Lambda}^{-1} \mathbf{Z}_2 \left(\frac{\partial \boldsymbol{\mu}_{t|t-1}}{\partial (\mathrm{vec}\,\mathbf{K})^{\top}}\right),$$

$$\mathcal{I}_t^{(\mathrm{v}(\mathbf{K}))}(\boldsymbol{\theta}) = \frac{\nu+R}{\nu+R+2}\left(\frac{\partial \boldsymbol{\mu}_{t|t-1}}{\partial (\mathrm{vec}\,\mathbf{K})^{\top}}\right)^{\top} \mathbf{Z}_2^{\top} \boldsymbol{\Lambda}^{-1} \mathbf{Z}_2 \left(\frac{\partial \boldsymbol{\mu}_{t|t-1}}{\partial (\mathrm{vec}\,\mathbf{K})^{\top}}\right).$$

S7 Simulation study

S7.1 Finite sample properties

To evaluate the finite sample properties of the maximum likelihood estimator, we ran an extensive simulation study, composed of six different cases, one for each parameter of interest. An overview of the simulation study is reported in Table S1. In each case study, we consider different values of the parameter of interest, while the other parameters are set to default values that are highlighted in bold in Table S1. For instance, in case study (i), we focus on ϕ that varies among $\{0, 0.4, 0.8\}$, while the other parameters are set to their default values. In the table, $\mathbf{1}_R$ denotes the R-dimensional vector of ones and $\boldsymbol{\kappa} = \{0.6, \mathbf{1.0}, 1.5\}\mathbf{1}_R$ means that the values of $\boldsymbol{\kappa}$ are constant across the regions and equal to $\kappa = 0.6$ (first scenario), $\kappa = 1.0$ (second scenario, default value in bold) and $\kappa = 1.5$ (third scenario). In all case studies presented in Table S1, a null intercept and null slope are considered.

We explore 20 different scenarios and, for each scenario, we simulate $R = 70$ time series, with length $T = 500$, under the spatial score driven model (4)-(7). The spatial weight matrices are assumed to be the same for the responses and the disturbances, i.e., $\mathbf{W}_1 = \mathbf{W}_2 = \mathbf{W}$ and defined as in Section 4.2, Figure 1. Such specific choices are done to mimic the features of the real dataset (see Section 4.1). Each scenario is then repeated 100 times.

Table S1: Simulation study design.

Case	Parameter	Values
(i)	ϕ	{0.0, 0.4, **0.8**}
(ii)	ρ_1	{0.0, 0.4, **0.8**}
(iii)	ρ_2	{0.0, **0.4**, 0.8}
(iv)	ν	{8, **15**, 50, 100, 300}
(v)	$\boldsymbol{\kappa}$	{0.6, **1.0**, 1.5} $\mathbf{1}_R$
(vi)	$\boldsymbol{\lambda}$	{−1.0, **−0.5**, 0.5} $\mathbf{1}_R$

All the simulation results are summarized in the following tables. Table S2 reports the estimated scalar parameters of the three scenarios evaluated in case (i), while Figure S2 shows the estimated $\boldsymbol{\lambda}$ and $\boldsymbol{\kappa}$ of case (i) in the left and right panel, respectively. In Table S3 we report the estimated scalar parameters of the three scenarios evaluated in case (ii), while

Figure S3 shows the estimated $\boldsymbol{\lambda}$ and $\boldsymbol{\kappa}$ of case (ii) in the left and right panel, respectively. In Table S4 we report the estimated scalar parameters of the three scenarios evaluated in case (iii), while Figure S4 shows the estimated $\boldsymbol{\lambda}$ and $\boldsymbol{\kappa}$ of case (iii) in the left and right panel, respectively. In Table S5 we report the estimated scalar parameters of the three scenarios evaluated in case (iv), while Figure S5 shows the estimated $\boldsymbol{\lambda}$ and $\boldsymbol{\kappa}$ of case (iv) in the left and right panel, respectively. In Table S6 we report the estimated scalar parameters of the three scenarios evaluated in case (v), while Figure S6 shows the estimated $\boldsymbol{\lambda}$ and $\boldsymbol{\kappa}$ of case (v) in the left and right panel, respectively. In Table S7 we report the estimated scalar parameters of the three scenarios evaluated in case (vi), while Figure S7 shows the estimated $\boldsymbol{\lambda}$ and $\boldsymbol{\kappa}$ of case (vi) in the left and right panel, respectively.

Overall, the true values of ϕ, ρ_1, ρ_2 and $\boldsymbol{\lambda}$ are always very well recovered, while the degrees of freedom parameter ν is typically slightly underestimated; however, its true value is always included in the 95% confidence interval. Conversely, the estimates of $\boldsymbol{\kappa}$ are sometimes moderately overestimated, with increasing precision as ν increases (see Figure S5). We remark here that, as the recursion for $\boldsymbol{\mu}_{t|t-1}$ has no intercept, due to the inclusion of fixed effects in the model, the initial value $\boldsymbol{\mu}_{1|0}$ is set equal to the null vector.

Table S2: Summary of the estimated scalar parameters in the three scenarios of case (i); standard errors are reported in brackets.

	True value	$\phi = 0$	$\phi = 0.4$	$\phi = 0.8$
ϕ	-	0.00 (0.03)	0.40 (0.03)	0.80 (0.01)
ρ_1	0.80	0.80 (0.01)	0.79 (0.07)	0.78 (0.08)
ρ_2	0.40	0.40 (0.02)	0.41 (0.07)	0.42 (0.09)
ν	15.00	13.28 (0.96)	13.29 (0.96)	13.31 (0.96)
β_0	0.00	0.00 (0.01)	0.00 (0.01)	0.00 (0.01)
β_1	0.00	0.00 (0.01)	0.00 (0.01)	0.00 (0.01)

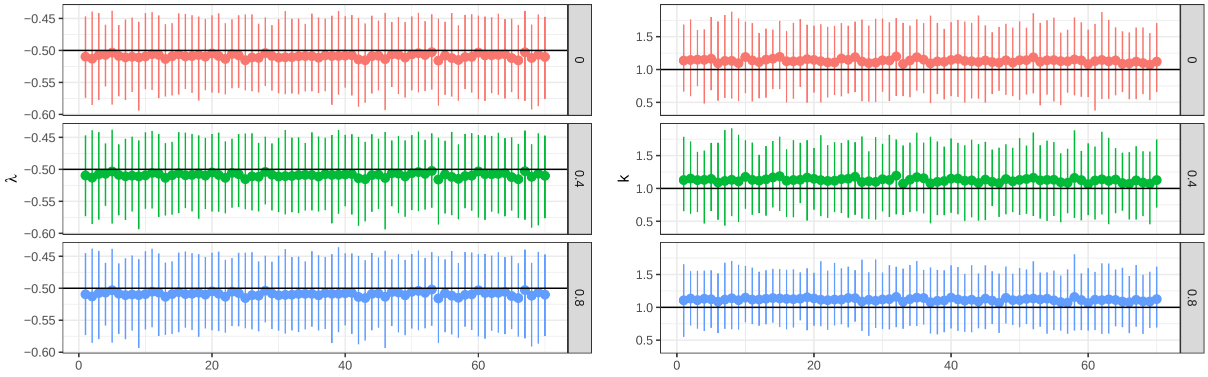

Figure S2: Estimated $\boldsymbol{\lambda}$ (left panel) and $\boldsymbol{\kappa}$ (right panel) in case (i). Dots represent the mean computed across the 100 simulated datasets, while the bars represent the 95% confidence interval. Solid black lines represent the true value.

Table S3: Summary of the estimated scalar parameters in the three scenarios of case (ii); standard errors are reported in brackets.

	True value	$\rho_1 = 0$	$\rho_1 = 0.4$	$\rho_1 = 0.8$
ϕ	0.80	0.80 (0.01)	0.80 (0.01)	0.80 (0.01)
ρ_1	-	0.00 (0.05)	0.39 (0.06)	0.78 (0.08)
ρ_2	0.40	0.40 (0.04)	0.40 (0.05)	0.42 (0.09)
ν	15.00	13.31 (0.96)	13.31 (0.96)	13.31 (0.96)
β_0	0.00	0.00 (0.01)	0.00 (0.01)	0.00 (0.01)
β_1	0.00	0.00 (0.01)	0.00 (0.01)	0.00 (0.01)

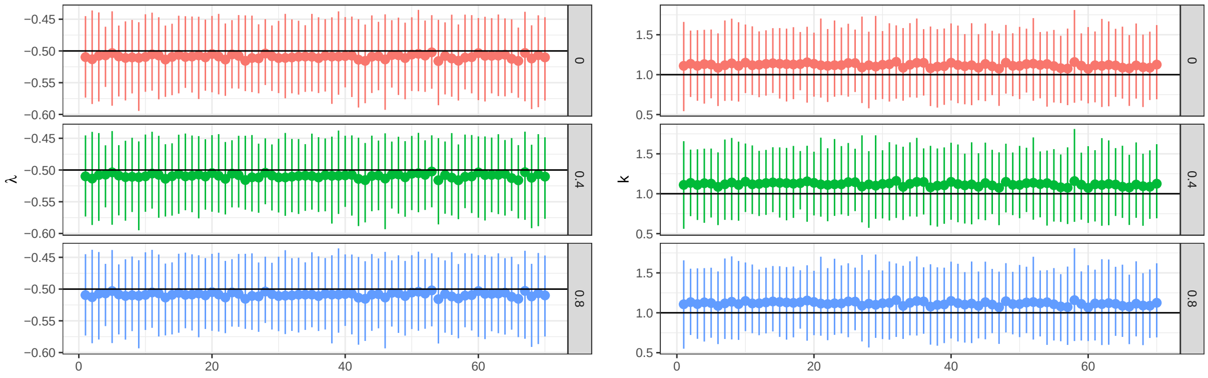

Figure S3: Estimated $\boldsymbol{\lambda}$ (left panel) and $\boldsymbol{\kappa}$ (right panel) in case (ii). Dots represent the mean computed across the 100 simulated datasets, while the bars represent the 95% confidence interval. Solid black lines represent the true value.

Table S4: Summary of the estimated scalar parameters in the three scenarios of case (iii); standard errors are reported in brackets.

	True value	$\rho_2 = 0$	$\rho_2 = 0.4$	$\rho_2 = 0.8$
ϕ	0.80	0.80 (0.01)	0.80 (0.01)	0.80 (0.01)
ρ_1	0.80	0.80 (0.01)	0.78 (0.08)	0.79 (0.03)
ρ_2	-	0.00 (0.02)	0.42 (0.09)	0.80 (0.03)
ν	15.00	13.31 (0.96)	13.31 (0.96)	13.31 (0.96)
β_0	0.00	0.00 (0.00)	0.00 (0.01)	0.00 (0.02)
β_1	0.00	0.00 (0.01)	0.00 (0.01)	0.00 (0.01)

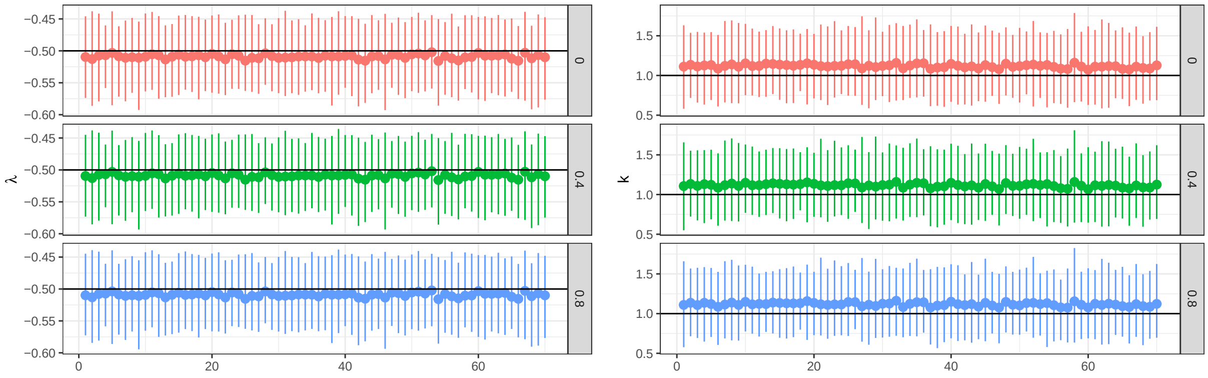

Figure S4: Estimated $\boldsymbol{\lambda}$ (left panel) and $\boldsymbol{\kappa}$ (right panel) in case (iii). Dots represent the mean computed across the 100 simulated datasets, while the bars represent the 95% confidence interval. Solid black lines represent the true value.

Table S5: Summary of the estimated scalar parameters in the five scenarios of case (iv); standard errors are reported in brackets.

	True value	$\nu = 8$	$\nu = 15$	$\nu = 50$	$\nu = 100$	$\nu = 300$
ϕ	0.80	0.80 (0.01)	0.80 (0.01)	0.80 (0.01)	0.80 (0.00)	0.80 (0.00)
ρ_1	0.80	0.78 (0.08)	0.78 (0.08)	0.80 (0.01)	0.80 (0.01)	0.80 (0.01)
ρ_2	0.40	0.42 (0.08)	0.42 (0.09)	0.40 (0.02)	0.40 (0.01)	0.40 (0.01)
ν	-	7.05 (0.45)	13.31 (0.96)	44.86 (4.18)	90.86 (10.63)	267.95 (46.68)
β_0	0.00	−0.00 (0.01)	−0.00 (0.01)	−0.00 (0.01)	−0.00 (0.01)	−0.00 (0.02)
β_1	0.00	0.00 (0.01)	0.00 (0.01)	−0.00 (0.01)	−0.00 (0.01)	−0.00 (0.01)

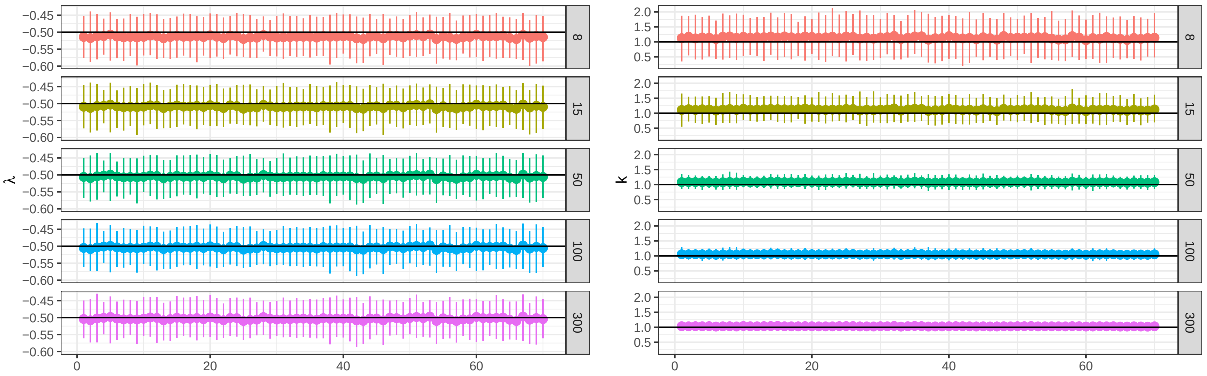

Figure S5: Estimated $\boldsymbol{\lambda}$ (left panle) and $\boldsymbol{\kappa}$ (right panel) in case (iv). Dots represent the mean computed across the 100 simulated datasets, while the bars represent the 95% confidence interval. Solid black lines represent the true value.

Table S6: Summary of the estimated scalar parameters in the five scenarios of case (v); standard errors are reported in brackets.

	True value	$\kappa = 0.6$	$\kappa = 1$	$\kappa = 1.5$
ϕ	0.80	0.80 (0.01)	0.80 (0.01)	0.80 (0.01)
ρ_1	0.80	0.75 (0.13)	0.78 (0.08)	0.78 (0.09)
ρ_2	0.40	0.45 (0.13)	0.42 (0.09)	0.42 (0.09)
ν	15.00	13.27 (0.95)	13.31 (0.96)	13.37 (0.97)
β_0	0.00	0.00 (0.01)	0.00 (0.01)	0.00 (0.01)
β_1	0.00	0.00 (0.01)	0.00 (0.01)	0.00 (0.01)

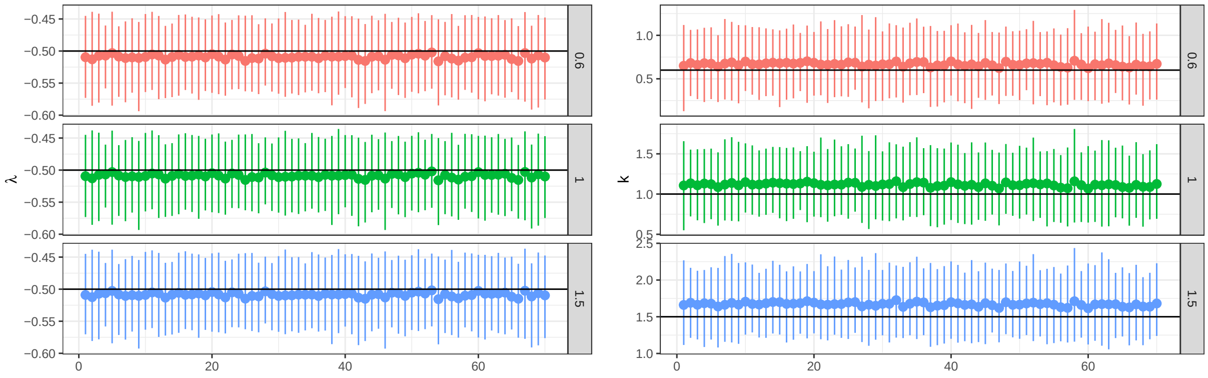

Figure S6: Estimated $\boldsymbol{\lambda}$ (left panel) and $\boldsymbol{\kappa}$ (right panel) in case (v). Dots represent the mean computed across the 100 simulated datasets, while the bars represent the 95% confidence interval. Solid black lines represent the true value.

Table S7: Summary of the estimated scalar parameters in the five scenarios of case (vi); standard errors are reported in brackets.

	True value	$\lambda = -1$	$\lambda = -0.5$	$\lambda = 0.5$
ϕ	0.80	0.80 (0.01)	0.80 (0.01)	0.80 (0.01)
ρ_1	0.80	0.80 (0.01)	0.78 (0.08)	0.79 (0.06)
ρ_2	0.40	0.40 (0.02)	0.42 (0.09)	0.41 (0.07)
ν	15.00	13.31 (0.96)	13.31 (0.96)	13.31 (0.96)
β_0	0.00	0.00 (0.00)	0.00 (0.01)	0.00 (0.02)
β_1	0.00	0.00 (0.00)	0.00 (0.01)	0.00 (0.02)

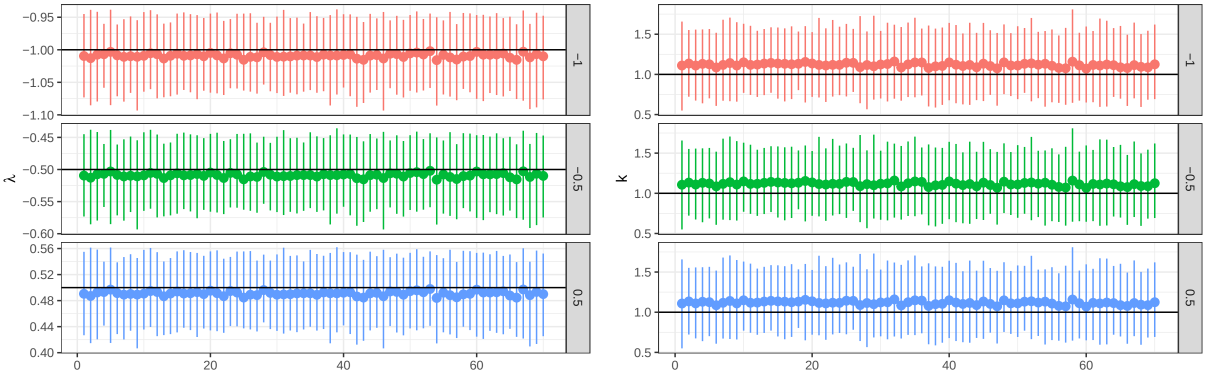

Figure S7: Estimated $\boldsymbol{\lambda}$ (left panel) and $\boldsymbol{\kappa}$ (right panel) in case (vi). Dots represent the mean computed across the 100 simulated datasets, while the bars represent the 95% confidence interval. Solid black lines represent the true value.

S7.2 Exogenous variables

In order to assess the finite sample properties of the MLEs when fixed effects are included in the proposed model, we conducted a simulation study considering two different exogenous variables. For each case, we analyze three different scenarios, one per each β_1, while the other parameters are set equal to their default values (see bold values in Table S1). For each scenario, we simulate $R = 70$ time series, with length $T = 500$, under the spatial score driven model with $\beta_0 = 0$ and $\mathbf{W}_1 = \mathbf{W}_2 = \mathbf{W}$ shown in Figure 1.

Firstly, we consider an exogenous variable that is designed to mimic a task-specific

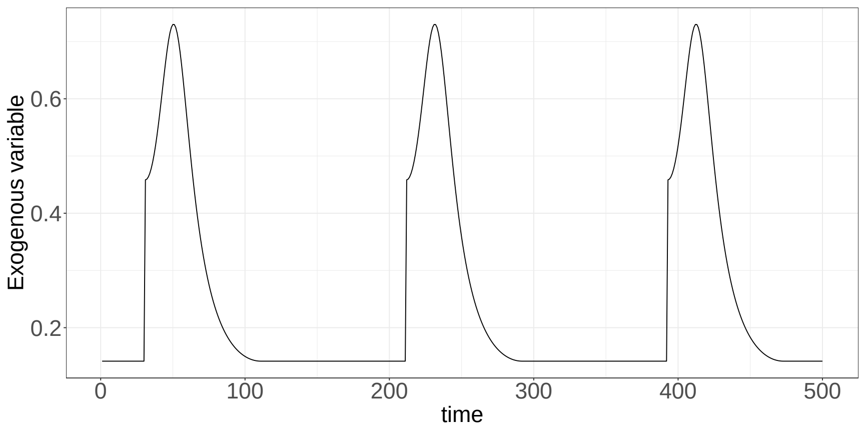

Figure S8: Exogenous variable to mimic a task-specific stimulus function.

stimulus function. Similar choices of the design matrix are common in the neuroimaging literature when dealing with task based analysis (Mejia et al., 2020). The exogenous variable is assumed to be the same across all different regions and is represented in Figure S8. We analyze three different scenarios, one per each β_1, $\beta_1 \in \{-0.5, 1, 2\}$; for each scenario we generate 100 different datasets. The estimates of the scalar parameters are reported in Table S8, while Figure S9 shows the estimated $\boldsymbol{\lambda}$ and $\boldsymbol{\kappa}$ in the left and right panel, respectively.

Table S8: Scalar estimates of the model when adjusting for the exogenous variable in Figure S8; standard errors are reported in brackets.

	True value	$\beta_1 = 0.5$	$\beta_1 = 1$	$\beta_1 = 2$
ϕ	0.80	0.80 (0.01)	0.80 (0.01)	0.80 (0.01)
ρ_1	0.80	0.79 (0.05)	0.80 (0.06)	0.82 (0.01)
ρ_2	0.40	0.41 (0.06)	0.40 (0.06)	0.37 (0.02)
ν	15.00	13.49 (0.96)	13.91 (0.98)	14.64 (1.03)
β_0	0.00	−0.01 (0.01)	−0.01 (0.01)	−0.01 (0.01)
β_1	-	0.53 (0.14)	1.01 (0.28)	1.78 (0.09)

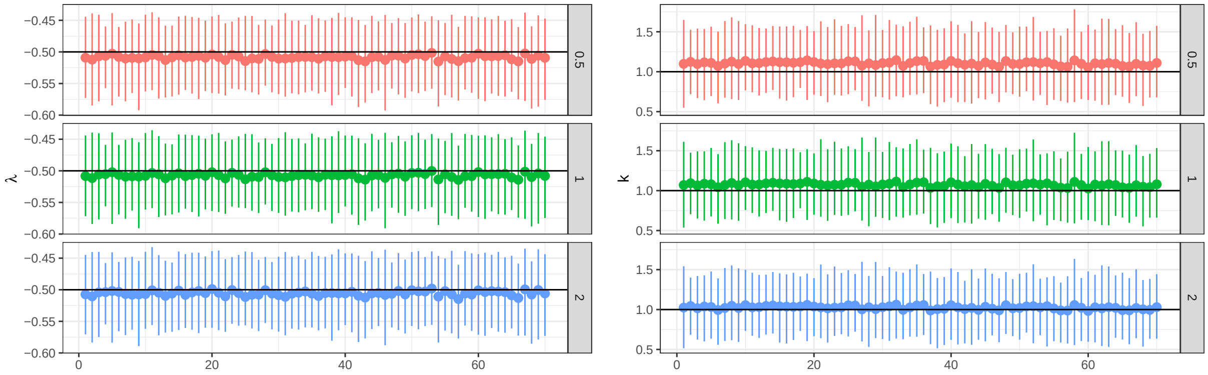

Figure S9: Estimated $\boldsymbol{\lambda}$ (left panel) and $\boldsymbol{\kappa}$ (right panel) when adjusting for the exogenous variable in Figure S8. Dots represent the mean computed across the 100 simulated datasets, while the bars represent the 95% confidence interval. Solid black lines represent the true value.

Secondly, we simulate an exogenous variable that has a sinusoidal shape, i.e., $x = 0.5\sin(t), t \in [0, \pi]$, that is shared across the 70 regions. Then, a random error from a normal distribution with zero mean and standard deviation equal to 0.01 is added to each of the 70 time series. In other words, this simulation case allows us to assess the finite sample properties of the MLE in presence of a region-specific exogenous variable. We analyze three different scenarios, one per each β_1, $\beta_1 \in \{-0.5, 0.2, 0.5\}$; for each scenario we generate 100 different datasets. The estimates of the scalar parameters are reported in Table S9, while Figure S10 shows the estimated $\boldsymbol{\lambda}$ and $\boldsymbol{\kappa}$ in the left and right panel, respectively.

In all scenarios of the two case studies, the parameters are well recovered, including the coefficient associated with the exogenous variables.

Table S9: Scalar estimates of the model when adjusting for the exogenous variable with sinusoidal shape; standard errors are reported in brackets.

	True value	$\beta_1 = -0.5$	$\beta_1 = 0.2$	$\beta_1 = 0.5$
ϕ	0.80	0.80 (0.01)	0.80 (0.01)	0.80 (0.01)
ρ_1	0.80	0.80 (0.01)	0.80 (0.01)	0.80 (0.01)
ρ_2	0.40	0.40 (0.02)	0.40 (0.02)	0.40 (0.02)
ν	15.00	13.32 (0.96)	13.31 (0.96)	13.31 (0.96)
β_0	0.00	0.00 (0.01)	0.00 (0.01)	0.00 (0.01)
β_1	-	-0.49 (0.04)	0.19 (0.04)	0.48 (0.04)

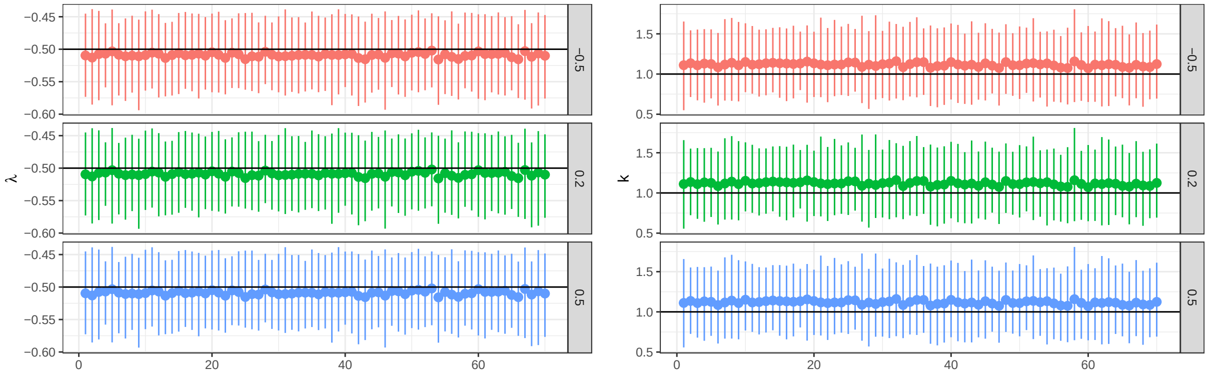

Figure S10: Estimated $\boldsymbol{\lambda}$ (left panel) and $\boldsymbol{\kappa}$ (right panel) when adjusting for the exogenous variable with sinusoidal shape. Dots represent the mean computed across the 100 simulated datasets, while the bars represent the 95% confidence interval. Solid black lines represent the true value.

S7.3 Potential misspecification

We carried out a simulation experiment to assess the goodness of fit of the spatial score driven model developed in the paper, denoted here in short as SARAR-t, when the data come from a dynamic SARAR model with Gaussian errors, denoted here in short as SARAR-N.

Specifically, we simulate from a SARAR-N with the parameters set to their default values and highlighted in Table S1, i.e., $\phi = 0.8$, $\rho_1 = 0.8$, $\rho_2 = 0.4$, $\beta_0 = \beta_1 = 0$, $\mathbf{K} = \mathbf{I}_R$ and $\mathbf{\Lambda} = -0.5\mathbf{I}_R$, which is equivalent to assume $\boldsymbol{\eta}_t \sim MVN(0, e^{-1}\mathbf{I}_R)$. Finally, we set $\mathbf{W}_1 = \mathbf{W}_2 = \mathbf{W}$ shown in Figure 1. We generate 100 datasets and we fit both the SARAR-t and the SARAR-N on each dataset.

The average and the standard deviation of the estimated parameters according to the two different models are identical up to the third decimal, except for ν that is defined only in SARAR-t model. It is important to mention that the average estimated ν is of the order of 10^5 with a with a median of $\hat{\nu} = 4,834$ and IQR equal to $14,310$, with a dispersed and long right tail empirical distribution. The average AIC and BIC for the two models are reported in Tabel S10 along with the associated 95% confidence interval. Evidently, the two specifications are equivalent, i.e., if the SARAR-t is fitted to Gaussian data, then, essentially, a Gaussian model is estimated.

Table S10: Average AIC and BIC for the two fitted models over the 100 datasets generated from a SARAR-N model. The 95% confidence interval is reported in brackets.

Model	AIC	BIC
SARAR-t	68,952 (68,496; 69,358)	70,188 (69,731; 70,594)
SARAR-N	68,952 (68,495; 69,359)	70,179 (69,722; 70,584)

S7.4 Computational details

For both simulations and real data analysis, we compute the maximum likelihood estimates via numerical optimization techniques that are suitable for nonlinear functions, by using the statistical software `R`. Specifically, we applied the PORT optimization routine (Gay, 1990) implemented in the `nlminb` function of the `stats` package. The running time for fitting the proposed model on 100 simulated datasets based on the default parameters in Table S1 is 5.40 hours on average, with a standard deviation of 0.43 hour, on one core of an Intel Xeon E7-8860 CPUs, with 2.2GHz clock speed. On the same workspace, the computational time for obtaining the analytical standard errors (Section S6) associated with each estimate on a single dataset is seven minutes, against the three hours required for computing the numerical ones.

S8 Real data analysis appendix

S8.1 Some exploratory analysis

As a preliminary analysis, we tested the multivariate normality assumption on each subject, by applying the Mardia (1980), Royston (1983) and Henze and Zirkler (1990) tests. According to all three approaches, the multinormality assumption is strongly violated (p-values ~ 0) for each subject. Testing the hypothesis of multivariate normality on the data is coherent with our setting in that, if the unconditional distribution of $\boldsymbol{y_t}$ were Gaussian, then the model would be linear and Gaussian, i.e., the conditional distribution would be Gaussian and so would be all the marginals. Normality tests on the marginals, at the ROI level for each subject, have been carried out in a previous study by Gasperoni and Luati (2018) and lead to the rejection of normality in 51% cases, with an average kurtosis equal to 4.0478 against an average skewness equal to 0.0085. We eventually come to the conclusion that a spatio-temporal model that assumes multinormality of the data would not be appropriate for the actual sample.

Figure S11 shows the Normal Q-Q plot of the data for subject 6 (left panel) and 13 (right panel). The plots support the conclusion that a spatio-temporal model that assumes multinormality of the data would not be appropriate, as we argue above. In fact, the patterns of deviation from the 45-degree line clearly highlight the heavy-tailed nature of the data and thus, thicker tails are required to accommodate such feature of the data. Moreover, both the panels also illustrate that there is no evidence of asymmetric behavior in the tails, which does not motivate the introduction of an additional skewness parameter in our parametric model.

As another graphical tool to obtain guidance on the choice of a proper multivariate distribution, we use the scatter plots displayed in Figure S12. In each panel, we observe strong clustering of several observations in the joint upper and lower quadrants, and therefore, the presence of positive tail dependence. Thus, large positive and negative outliers tend to be highly correlated, which further motivates our proposal for a parsimonious nonlinear model based on the multivariate Student-t distribution.

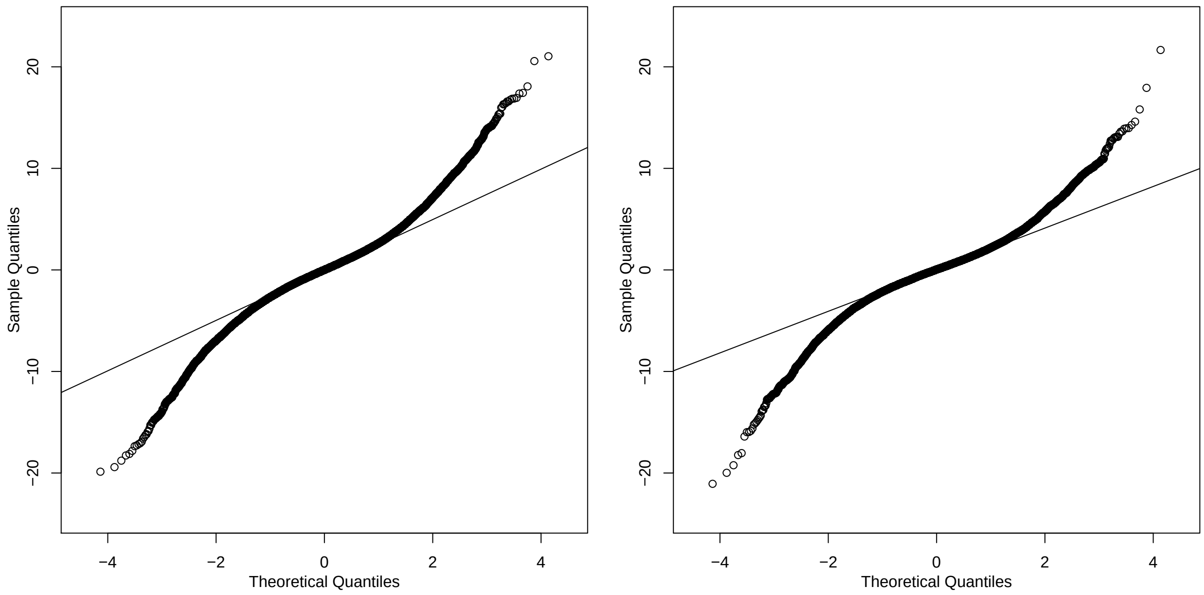

Figure S11: Normal Q-Q plot of the data for subject 6 (left) and subject 13 (right).

S8.2 More on estimation results

Figures S13 and S14 display the analytic standard errors associated with the estimated parameters $\exp\{\boldsymbol{\lambda}\}$ and $\boldsymbol{\kappa}$ for subjects 6 and 13 presented in Figure 2 and 3 of the manuscript, respectively.

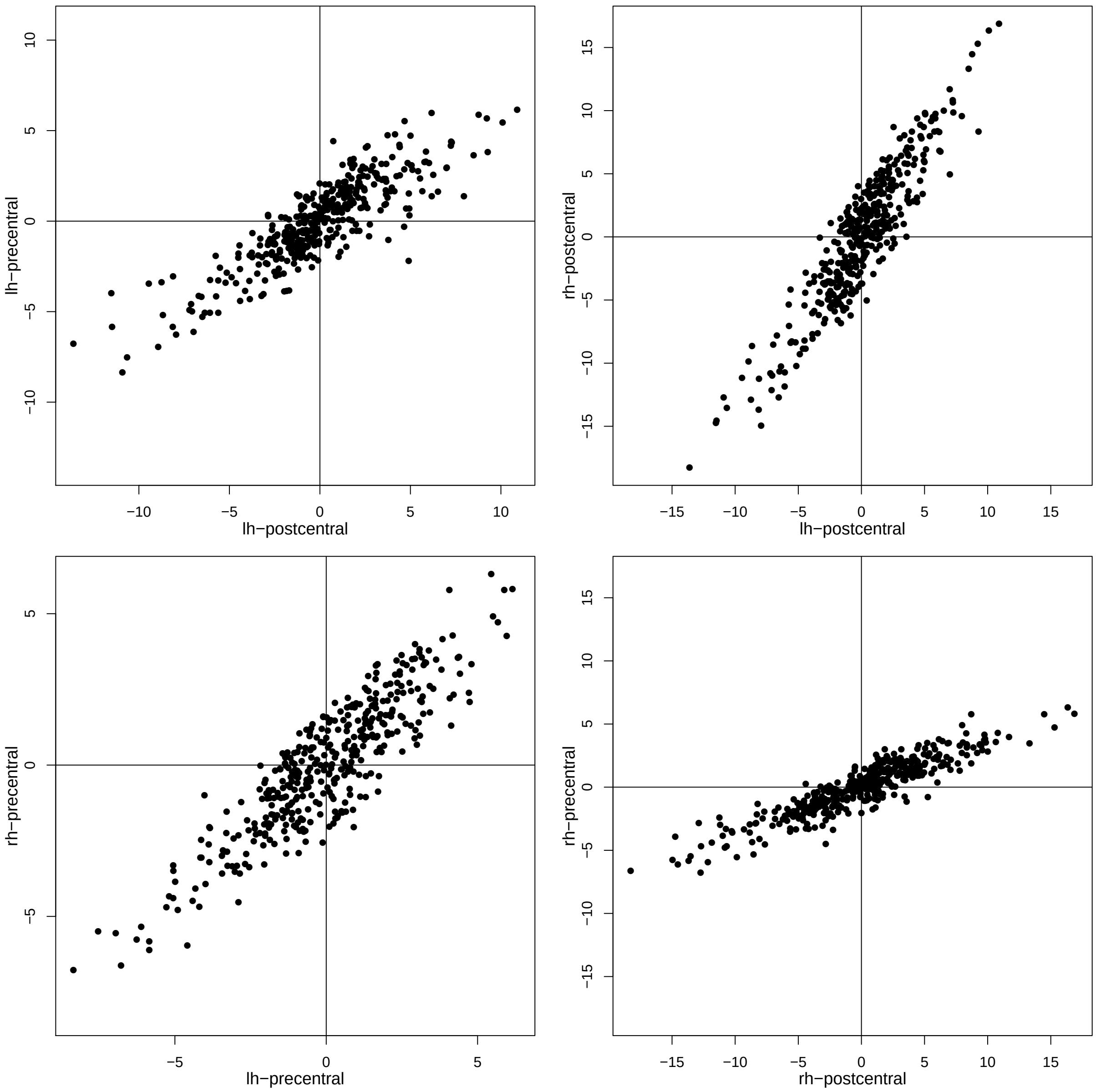

Figure S12: Scatterplot of ROIs `postcentral` and `precentral` in the left (`lh`) and right (`rh`) hemispheres.

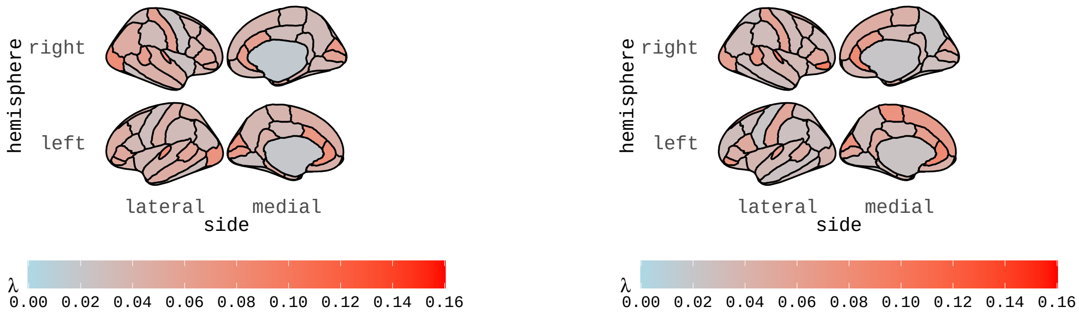

Figure S13: Standard errors of the estimated $\exp\{\boldsymbol{\lambda}\}$ for subject 6 (left panel) and subject 13 (right panel).

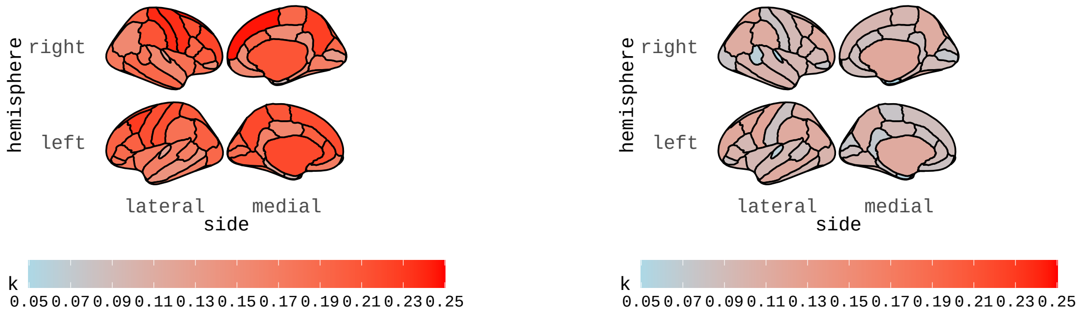

Figure S14: Standard errors of the estimated $\boldsymbol{\kappa}$ for subject 6 (left panel) and subject 13 (right panel).

S8.3 Diagnostics

As a first diagnostic tool, we illustrate the in-sample prediction accuracy of the proposed spatial score driven model. Specifically, we provide 95% prediction intervals (PIs) for $\{\boldsymbol{y}_t\}_{t=1}^{T}$, with $T = 404$, based on the empirical parameter estimates obtained for the two subjects, and report the proportion of times the observed data are inside the PIs. To calculate the in-sample forecast bands we follow the simulation-based strategy discussed in Blasques et al. (2016, Section 3.3).

The overall empirical coverage is 92.7% and 93.7% for subject 6 and 13, respectively, showing the relatively good prediction accuracy of the model. Figure S15 illustrates the empirical coverage of 95% PIs computed over time for subject 6 and 13. The plots evidence

that, at some specific time-points, the coverage may be quite low. A similar pattern is observed when the coverage is averaged over time and displayed at the brain regions. We argue that the low coverage at specific regions or times is related to outlying observations, that, in our framework, are interpreted as spontaneous activations, see Figure 5 in the main document. To support this argument, note that, in Figure S15, points are reproduced with color intensities that represent the percentage of regions where spontaneous activations were detected. It is evident that the time-points where the coverage is low typically correspond to times where spontaneous activations (outlying values) have been detected in several regions (red and purple dots). We remark that we have considered as activations those residual values that indicate an *excess* with respect to the average blood oxygenation level dependent signal. One can envisage outlying observations also in the left tail of the residuals distribution, possibly related to the (debated issue of) post-stimulus, or post-activation, signal undershoot, see van Zijl et al. (2012). Taking into account also negative outlying values explains the few blue dots with low coverage.

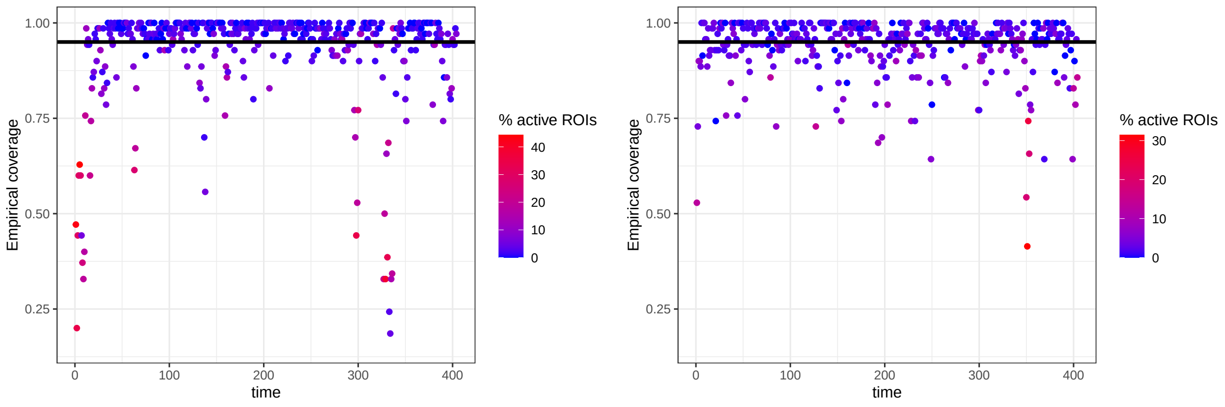

Figure S15: Empirical coverage of 95% PIs for each time of subject 6 (left panel) and subject 13 (right panel); color intensities are related to the percentage of brain regions where spontaneous activations were detected at each time.

Figure S15 not only allows us to test whether the empirical coverages are significantly higher or lower than the 95% level, but also permit to assess the goodness of fit of the dynamic features of the model and, in principle, visually detect if there are volatility clusters, that may not be directly observed in the original data. If present, volatility clusters would indicate a dynamic conditional variance, that the model is not designed to capture. To provide statistical evidence of the fact that the constant volatility assumption is

consistent with the data analyzed in the paper, we performed the m-th order autoregressive conditional heteroskedasticity (ARCH) test described in Juselius (2006, page 74). Results for $m = 1, \ldots, 4$ show that no residuals exhibit significant ARCH effects and thus we may conclude that the assumption of constant volatility is consistent with the data.

As far as the spatial dependence is concerned, we used the permutation test for Moran's I statistic (Cliff and Ord, 1981) to assess residual spatial autocorrelation at each time t. The null hypothesis of spatial randomness in the residuals is rejected only in the 6% and 2% of time-points for subject 6 and 13, respectively. In other words, the SAR score driven model captures most of the spatial dependence structure in the R-fMRI data.

To determine the suitability of the multivariate Student-t assumption, we performed, on the estimated spatially filtered residuals, the one-sided Lagrange Multiplier (LM) test developed by Fiorentini et al. (2003, page 537) for the null hypothesis of multivariate Normal residuals versus the alternative of a multivariate Student-t distribution. Results indicate a strong rejection of the null hypothesis (p-values ~ 0) and validate the assumption of the Student-t distribution for the error term. Assessing the hypothesis of a multivariate Student-t distribution is yet a challenging task, in particular when a high number of variables is considered. Alternative tests to the one we performed are the non-parametric test developed by Justel et al. (1997), based on Rosenblatt's transformation and primarily indicated when the number of time series involved is small, the likelihood-based test developed by Mencia and Sentana (2012) of a multivariate Student-t or Normal distribution with respect to the alternative Generalized Hyperbolic distribution, and the variant by Amengual and Sentana (2020) for moderately large dimensions ($R = 10$).

In practical applications, departure from normality is often assessed based on excess kurtosis and asymmetry. With this aim, to further examine the adequacy of the multivariate Student-t distribution versus the alternative of a multivariate Normal distribution, Figure S16, reports the normal Q-Q plot of the estimated spatial prediction error $\hat{\mathbf{Z}}_1 \hat{\boldsymbol{v}}_t$ for subject 6 (left panel) and subject 13 (right panel). The aim is to visually compare the sample quantiles of $\hat{\mathbf{Z}}_1 \hat{\boldsymbol{v}}_t$ against the theoretical quantiles of multivariate normal data. As the points are not scattered closely around the 45-degree line, we conclude that the multivariate Normal distribution does not explain the heavy tails observed in the spatial prediction error series and therefore does not provide an appropriate modeling approach.

As a final check, we further validate the use of the conditional Student-t distribution by using simulated data from the fitted SAR model. Figure S17 shows the Q-Q plot of the simulated data using the estimated parameters for subject 6 (left panel) and 13 (right panel). The plots look very close to the ones based on the observed R-fMRI data and displayed in Figure S11. Hence, we conclude that the proposed model fits the data well.

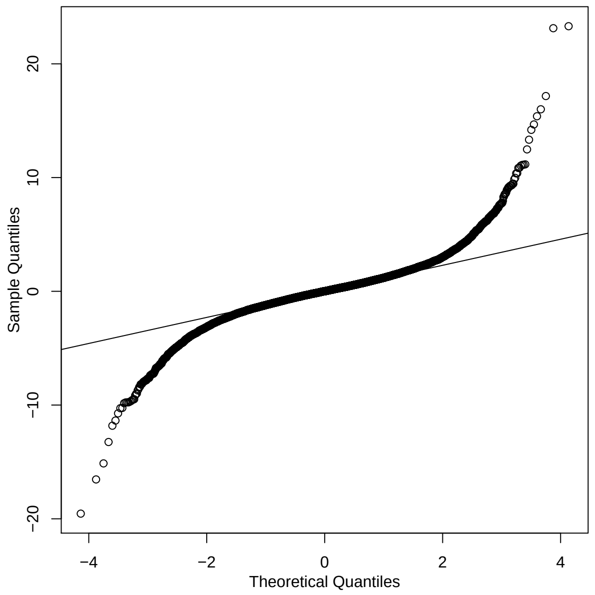

Figure S16: Normal Q-Q plot of the estimated spatial prediction error $\hat{\mathbf{Z}}_1 \hat{\boldsymbol{v}}_t$ for subject 6 (left panel) and subject 13 (right panel).

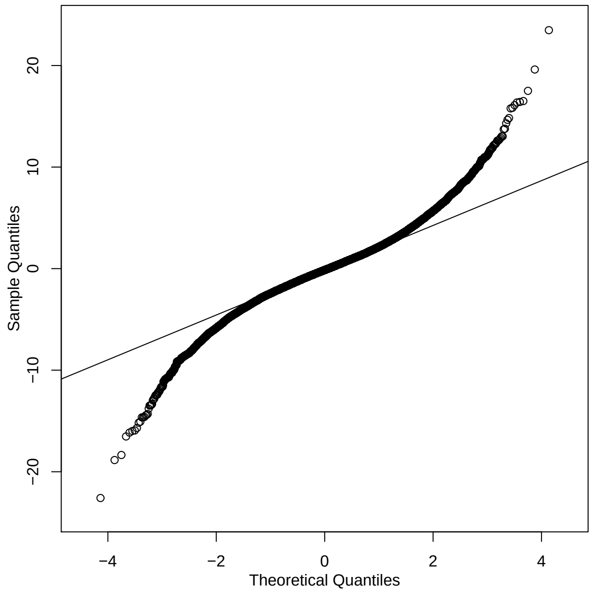

Figure S17: Normal Q-Q plot of data simulated from the estimated model for subject 6 (left panel) and subject 13 (right panel).

References

Abadir, K. M., R. D. H. Heijmans, and J. R. Magnus (2018). *Statistics.* Econometric Exercises. Cambridge University Press.

Amengual, D. and E. Sentana (2020). Is a normal copula the right copula? *Journal of Business & Economic Statistics 38*(2), 350–366.

Blasques, F., S. J. Koopman, K. Łasak, and A. Lucas (2016). In-sample confidence bands and out-of-sample forecast bands for time-varying parameters in observation-driven models. *International Journal of Forecasting 32*(3), 875–887.

Bollerslev, T. and J. M. Wooldridge (1992). Quasi-maximum likelihood estimation and inference in dynamic models with time-varying covariances. *Econometric Reviews 11*(2), 143–172.

Bougerol, P. (1993). Kalman filtering with random coefficients and contractions. *SIAM Journal on Control and Optimization 31*(4), 942–959.

Chu, J. T. (1956). Errors in normal approximations to the t, τ, and similar types of distribution. *Annals of Mathematical Statistics 27*(3), 780–789.

Cliff, A. D. and J. K. Ord (1981). *Spatial processes: models and applications.* London: Pion.

Crowder, M. J. (1976). Maximum likelihood estimation for dependent observations. *Journal of the Royal Statistical Society: Series B (Methodological) 38*(1), 45–53.

Dickey, J. M. (1967). Expansions of t densities and related complete integrals. *The Annals of Mathematical Statistics 38*(2), 503 – 510.

D'Innocenzo, E., A. Luati, and M. Mazzocchi (2020). A robust score driven filter for multivariate time series. *arXiv:2009.01517*.

Fang, K., S. Kotz, and K. Ng (1990). *Symmetric multivariate and related distributions.* Number 36 in Monographs on statistics and applied probability. Chapman & Hall.

Fiorentini, G., E. Sentana, and G. Calzolari (2003). Maximum likelihood estimation and inference in multivariate conditionally heteroscedastic dynamic regression models with Student t innovations. *Journal of Business & Economic Statistics 21*(4), 532–546.

Fisher, R. (1925). Expansions of “Student’s” integral in power of n^{-1}. *Metron 5*, 109–120.

Gasperoni, F. and A. Luati (2018). Robust methods for detecting spontaneous activations in fMRI data. In A. Canale, D. Durante, L. Paci, and B. Scarpa (Eds.), *Studies in Neural Data Science*, pp. 91–110. Springer.

Gay, D. M. (1990). Usage summary for selected optimization routines. *Computing science technical report 153*, 1–21.

Harvey, A. C. (2013). *Dynamic Models for Volatility and Heavy Tails: With Applications to Financial and Economic Time Series.* Cambridge University Press.

Henze, N. and B. Zirkler (1990). A class of invariant consistent tests for multivariate normality. *Communications in statistics-Theory and Methods 19*(10), 3595–3617.

Juselius, K. (2006). *The Cointegrated VAR Model: Methodology and Applications.* Oxford University Press.

Justel, A., D. Pena, and R. Zamar (1997). A multivariate Kolmogorov-Smirnov test of goodness of fit. *Statistics & Probability Letters 35*(3), 251–259.

Kotz, S. and S. Nadarajah (2004). *Multivariate t-distributions and their applications.* Cambridge University Press.

Krengel, U. and A. Brunel (1985). *Ergodic Theorems.* De Gruyter studies in mathematics. De Gruyter.

Mardia, K. (1980). 9 tests of univariate and multivariate normality. In *Analysis of Variance*, Volume 1 of *Handbook of Statistics*, pp. 279–320. Elsevier.

Mejia, A. F., Y. R. Yue, D. Bolin, F. Lindgren, and M. A. Lindquist (2020). A Bayesian general linear modeling approach to cortical surface fMRI data analysis. *Journal of the American Statistical Association 115*(530), 501–520.

Mencia, J. and E. Sentana (2012). Distributional tests in multivariate dynamic models with normal and Student-t innovations. *The Review of Economics and Statistics 94*(1), 133–152.

Royston, J. (1983). Some techniques for assessing multivariate normality based on the Shapiro-Wilk W. *Journal of the Royal Statistical Society: Series C (Applied Statistics) 32*(2), 121–133.

Straumann, D. and T. Mikosch (2006). Quasi-maximum-likelihood estimation in conditionally heteroscedastic time series: A stochastic recurrence equations approach. *The Annals of Statistics 34*(5), 2449–2495.

Tiao, G. C. and A. Zellner (1964). Bayes's theorem and the use of prior knowledge in regression analysis. *Biometrika 51*(1/2), 219–230.

Tricomi, F. G. and A. Erdelyi (1951). The asymptotic expansion of a ratio of gamma functions. *Pacific Journal of Mathematics 1*(1), 133–142.

van der Vaart, A. (1998). *Asymptotic Statistics*. Cambridge series on statistical and probabilistic mathematics. Cambridge University Press.

van Zijl, P. C., J. Hua, and H. Lu (2012). The BOLD post-stimulus undershoot, one of the most debated issues in fMRI. *NeuroImage 62*(2), 1092–1102.

Villa, C. and S. G. Walker (2014). Objective prior for the number of degrees of freedom of a t distribution. *Bayesian Analysis 9*(1), 197–220.

White, H. (1994). *Estimation, inference and specification analysis.* Econometric Society Monographs. Cambridge University Press.

White, H. (2001). *Asymptotic theory for econometricians.* Economic Theory, Econometrics, and Mathematical Economics. Emerald Group Publishing Limited.
